# Unveiling the Catalytic Mechanism of a Processive Metalloaminopeptidase

**DOI:** 10.1021/acs.biochem.3c00420

**Published:** 2023-11-04

**Authors:** Martha Clementine Simpson, Christopher John Harding, Ricardo Melo Czekster, Laura Remmel, Bela E. Bode, Clarissa Melo Czekster

**Affiliations:** † School of Biology, University of St Andrews, North Haugh, Biomolecular Sciences Building, KY16 9ST, Saint Andrews, United Kingdom; ‡ School of Computer Science and Digital Technologies, Department of Software Engineering and Cybersecurity, Aston University, B4 7ET, Birmingham,United Kingdom; § School of Chemistry, University of St Andrews, North Haugh, Purdie Building, KY16 9ST, Saint Andrews , United Kingdom

## Abstract

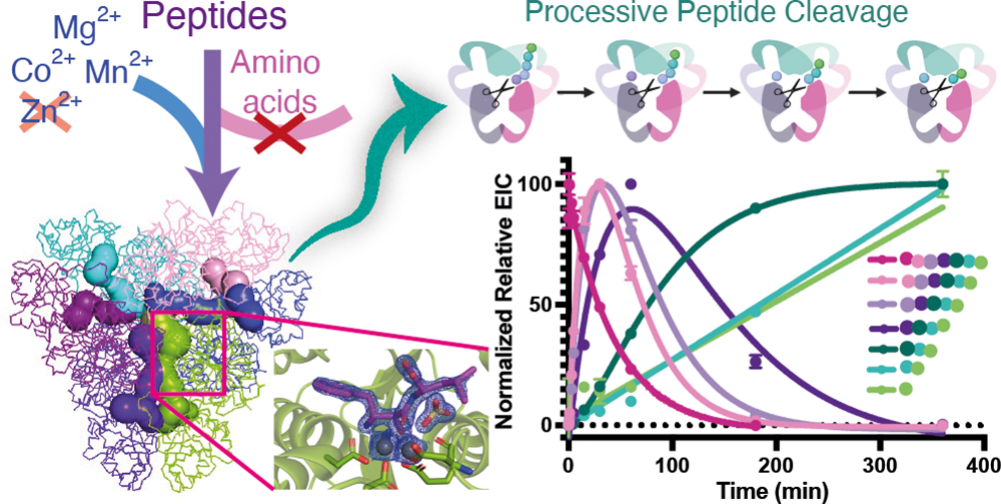

Intracellular leucine aminopeptidases (PepA) are metalloproteases from the family M17. These enzymes catalyze peptide bond cleavage, removing N-terminal residues from peptide and protein substrates, with consequences for protein homeostasis and quality control. While general mechanistic studies using model substrates have been conducted on PepA enzymes from various organisms, specific information about their substrate preferences and promiscuity, choice of metal, activation mechanisms, and the steps that limit steady-state turnover remain unexplored. Here, we dissected the catalytic and chemical mechanisms of *Pa*PepA: a leucine aminopeptidase from *Pseudomonas aeruginosa*. Cleavage assays using peptides and small-molecule substrate mimics allowed us to propose a mechanism for catalysis. Steady-state and pre-steady-state kinetics, pH rate profiles, solvent kinetic isotope effects, and biophysical techniques were used to evaluate metal binding and activation. This revealed that metal binding to a tight affinity site is insufficient for enzyme activity; binding to a weaker affinity site is essential for catalysis. Progress curves for peptide hydrolysis and crystal structures of free and inhibitor-bound *Pa*PepA revealed that *Pa*PepA cleaves peptide substrates in a processive manner. We propose three distinct modes for activity regulation: tight packing of *Pa*PepA in a hexameric assembly controls substrate length and reaction processivity; the product leucine acts as an inhibitor, and the high concentration of metal ions required for activation limits catalytic turnover. Our work uncovers catalysis by a metalloaminopeptidase, revealing the intricacies of metal activation and substrate selection. This will pave the way for a deeper understanding of metalloenzymes and processive peptidases/proteases.

## Introduction

Bacteria must adapt and respond to constant changes in the environment. Protein degradation is a crucial and irreversible cellular process necessary for the adaptation and survival of bacteria in response to environmental changes.^1^ Proteolysis of damaged proteins is a vital quality control process, with implications in cell signaling, sporulation, and biofilm dynamics.^2^ In bacteria, the N-termini of proteins are also important in modulating protein stability and half-life. Proteins fated for destruction possess destabilizing signals known as degrons, which may be added or removed according to cellular requirements.^3−5^

**Scheme 1:**
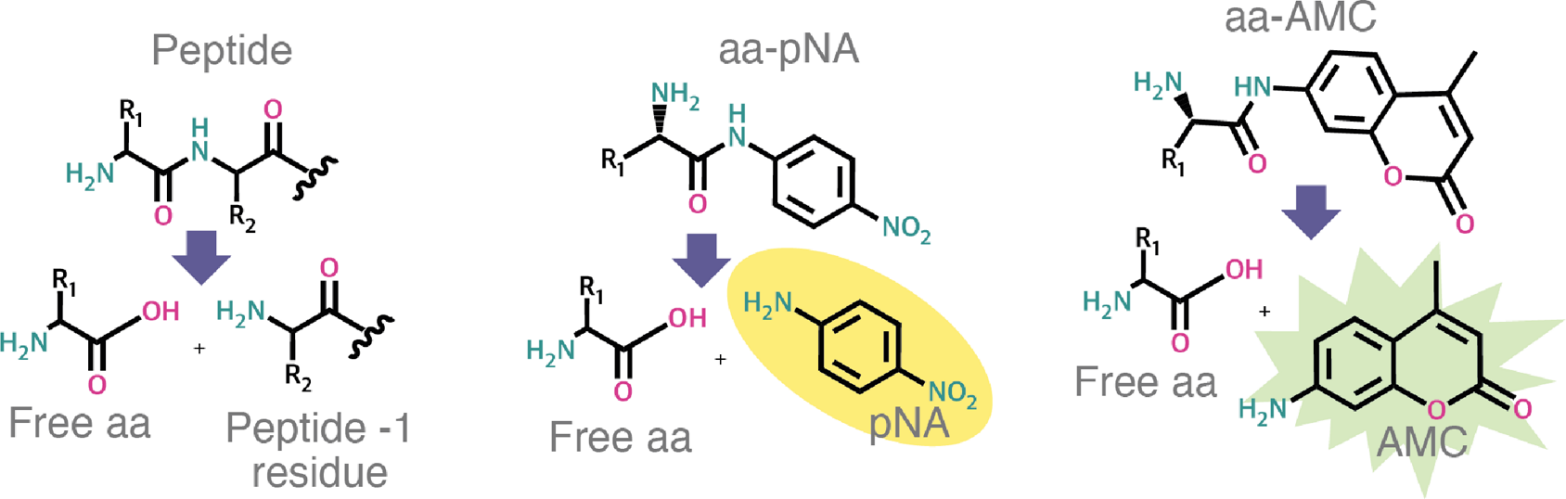
Substrates used by *Pa*PepA Left, cleavage of generic peptide substrate; middle, cleavage of amino acid-paranitroanilide (pNA shown as a yellow oval product that absorbs at 405 nm); right, cleavage of amino acid-7-amido-4-methylcoumarin (AMC shown as a green star fluorescent product with excitation: 341 nM, emission: 441 nM). R_1_ and R_2_ = amino acid side chains for different amino acids.

Mechanisms controlling the establishment and removal of degradative signals have not been well characterized. In some cases, specific amino acids may be added or removed to generate degrons. Aminopeptidases can trim N-terminal residues from target proteins and peptides, either rescuing them from degradation or exposing destabilizing residues that drive degradation forward. N-Terminal leucine is considered a destabilizing residue, leading to decreased protein half-life.^6^ Leucine aminopeptidases (PepA) are intracellular metalloenzymes that require Zn^2+^ and/or Zn^2+^ and Mn^2+^ for activation. The leucine aminopeptidase classification and inferred substrate preference were determined using model peptide substrates most commonly containing a chromophore leaving group. Substrates tested are shown in Scheme 1.

PepA enzymes from different organisms show variation in their substrate preferences and metal activation.^7−11^ We set out to dissect the kinetic mechanism, structure, metal ion dependence, and substrate selectivity of PepA from *Pseudomonas aeruginosa* (*Pa*PepA, UniProt Q02RY8). *P. aeruginosa* is an exceptionally adaptable organism, successfully colonizing soil, water, human skin, and lungs, as well as plants. In a laboratory setting, *P. aeruginosa* can survive in a growth-arrested state for months—even years—in distilled or salt water.^12,13^ Under conditions of nutrient scarcity, bacterial replication halts and metabolism shifts toward a state of survival and low energy expenditure.^6^ Protein recycling and quality control are likely to play crucial roles in these states. The role of *Pa*PepA in pathogenicity is also of interest. Transposon insertion and subsequent inactivation of PepA are associated with attenuation of *Pseudomonas* virulence in rat models of chronic respiratory infection.^14^ Furthermore, *Pa*PepA is implicated in regulation of alginate biosynthesis; overexpression of this exopolysaccharide is characteristic of *P. aeruginosa* infections.^11^ Mutations in *Pa*PepA have also been shown to increase antimicrobial resistance to certain antibiotics.^15^ Understanding the molecular determinants regulating peptidase activity is therefore of paramount importance, as this will advance our ability to characterize,^16^ design,^17^ and inhibit^18^ metalloenzymes.

Here, we dissected the kinetics of metal ion activation by *Pa*PepA. Steady-state and pre-steady-state enzymatic assays, solvent kinetic isotope effects, viscosity studies, isothermal titration calorimetry, electron paramagnetic resonance spectroscopy, and crystallography allowed us to propose a model for *Pa*PepA activation that relies heavily on metal availability and activation. Further modulation of PepA activity occurs with the nature of the available substrates and the presence of free amino acids. Chemistry limits the *Pa*PepA-catalyzed reaction during steady-state turnover.

## Materials and Methods

### Chemicals and General Methods

Buffers, salts, and common chemicals used in this investigation were purchased from Fisher Scientific and Merck. Peptides tested as substrates were commercially obtained from Peptide Synthetics. These peptides were obtained at >90% purity (by HPLC). Peptide identity was confirmed by using mass spectrometry. Analysis was carried out using either a 100 Å 4.6 mm × 50 mm column or a Kinetex XB-C18 2.6 μm 100 Å column, with a gradient moving from 0 to 80% acetonitrile over 8 min at 60 °C, a flow rate of 1.5 mL/min, and an injection volume of 20 μL. Peptides were purified in acetonitrile and water containing 0.1% trifluoroacetic acid prior to lyophilization. Bestatin was purchased from Fisher; peptides KA-AMC, RYLGYL (α-casein(90–95), and DRVYIHPF were purchased from Bachem, and MassPREP ADH Digestion Standard was from Waters. Primers and DNA fragments used for cloning were from IDT. Primer sequences are available on Table S1. All data analysis except for pre-steady-state data was carried out using GraphPad Prism 9.3.1 for Windows, GraphPad Software, San Diego, California USA, or SigmaPlot 14.5 (Systat Software, San Jose, California). Pre-steady-state data were analyzed using Kintek Global Explorer (KinTek Corporation, Snow Shoe, Pennsylvania).^19^

### Cloning, Expression, and Purification of *Pa*PepA

PepA from *P. aeruginosa* strain PA14 (*Pa*PepA, PA14_14470, UniProt: Q02RY8) was synthesized as a codon-optimized gBlock (IDT). The *Pa*PepA gene was inserted into a pJ414 expression plasmid to encode an N-terminal His_6_ tag, followed by a linker and a TEV protease cleavage site. Cloning was performed using Gibson Assembly^20^ with the NEBuilder HiFi DNA Assembly kit and commercially available *Escherichia coli* (*E. coli*) DH5α cells (NEB). The presence and identity of the desired gene were confirmed through sequencing (Eurofins).

Purified plasmid was transformed into *E. coli* BL21(DE3) cells (NEB) allowing heterologous expression of *Pa*PepA. Cells were cultured at 37 °C until an OD_600_ of 0.6–0.8 was reached. IPTG was added to a final concentration of 1 mM for induction. Subsequently, cells were grown overnight at 25 °C. Bacterial pellet from 6 L of culture was reconstituted in 180 mL of lysis buffer (50 mM HEPES, 250 mM NaCl, 10 mM imidazole, and 500 μM MnCl_2_, pH 7.5). Lysozyme (10 mg) and DNase (1 mg) were added. The solution was stirred for 30 min at 4 °C. Resuspended cells were lysed using a cell disrupter and then centrifuged for 30 min at 50,000*g* to separate soluble and insoluble fractions. Soluble components were filtered and loaded onto a 5 mL HisTrap FF nickel column (GE Healthcare), which had been pre-equilibrated with lysis buffer. *Pa*PepA was eluted from the column using elution buffer (50 mM HEPES, 250 mM NaCl, 250 mM imidazole, and 500 μM MnCl_2_, pH 7.5).

Elution fractions were pooled. A small fraction of the protein was removed and dialyzed (in 50 mM HEPES, 250 mM NaCl, pH 7.5), concentrated using Millipore Amicon Ultra-4 centrifugal filter units with 10,000 Da molecular weight cutoffs to 12 mg mL^–1^ and later used for crystallographic studies. TEV-cleaved protein was recalcitrant to crystallization efforts.

TEV protease was added to the remaining protein at a final ratio of 1:100 mg TEV:PepA. The protein was dialyzed (into 50 mM HEPES, 250 mM NaCl, and 500 μM MnCl_2_ pH 7.5) for 48 h. The cleaved protein was separated from the residual fusion protein using a second passage through the HisTrap column. The protein was dialyzed (in 50 mM HEPES, 250 mM NaCl, and 5 mM EDTA pH 7.5) for 2 h to remove MnCl_2 and_ then into 50 mM HEPES, 250 mM NaCl, pH 7.5, for a further 2 h to remove EDTA. The protein was concentrated to ∼8 mg mL^–1^ and flash frozen for further experiments.

### Crystallization Conditions

Uncleaved *Pa*PepA at 12 mg/mL in 50 mM HEPES, 250 mM NaCl, and pH 7.5 was used for crystallography studies. Crystals were formed at 20 °C using the sitting drop technique. Screen optimization resulted in the growth of apo enzyme crystals in 14% PEG 3350 and 200 mM ammonium nitrate, at a 2:1 ratio of protein:reservoir condition. 12 mg/mL of *Pa*PepA was also cocrystallized with 5 mM MnCl_2_ in 20% PEG 3350, and 172 mM ammonium nitrate, at a 1:1 ratio of protein:reservoir condition. For studies of *Pa*PepA bound to bestatin, 8 mg/mL *Pa*PepA was cocrystallized with 3 mM bestatin and 1 mM MnCl_2_, in 20% PEG 3350 and 254 mM ammonium nitrate at a 2:1 ratio of protein:reservoir condition.

Prior to fishing, crystals were cryoprotected with 2 μL of 20% ethylene glycol, 80% screen condition ±5 mM MnCl_2_, or ±3 mM bestatin and 1 mM MnCl_2_ according to whether the crystal to be fished was apoenzyme, metal-bound, or bestatin-bound.

Diffraction data were collected at the Diamond Light Source in Oxford, UK. Data reduction and processing was completed using XDS and the *xia2* suite. The structure was solved by molecular replacement with PHASER^21^ using the structure of *Pseudomonas putida* leucine aminopeptidase (*Pp*LAP) (PDB: 3H8F) as a search model. Protein structures were built/modified using COOT,^22^ with cycles of refinement in Phenix.^23,24^ Crystallographic data are listed in Table S10.

### Activity Assays

A BMG Labtech POLARstar Omega plate reader was used for activity assays and kinetic studies involving amino acids conjugated to para-nitroaniline (pNA) (Sigma) or peptides as substrates. Assays were carried out at 25 °C in clear half-area 96-well plates (Greiner Bio-One). The pNA product formed upon *Pa*PepA-mediated bond cleavage strongly absorbs at 405 nm; therefore, initial rates were measured by monitoring the increase in absorbance at 405 nm over time. For kinetic assays investigating Leu-AMC as a substrate, a SpectraMax M2E was used. Reactions were carried out using black half-area 96-well plates with clear bottoms (Greiner Bio-One). Product formation was assessed by monitoring changes in fluorescence, with the excitation wavelength set at 341 nm and emission at 441 nm. The cutoff was 420 nm. PMT gain was set to medium, with six flashes per read. *Pa*PepA was added at a final concentration of 100 nM to start reactions. Calibration curves of pNA concentration vs absorbance and AMC vs fluorescence were constructed, and initial rate values were divided by the slope of these calibration curves to reveal the rate of product formation over time (Table S2).

For all assays excluding pH dependence curves, buffer conditions were as follows: 100 mM HEPES, 50 mM KCl, and pH 8.0. For assays carried out under pseudo-first-order conditions, one reaction component was fixed and in excess, while the other was varied. If [metal] was varied, [Leu-pNA] was fixed at 2 mM or [Leu-AMC] was fixed at 170 μM. For assays involving variation of aa-pNA or aa-AMC, MnCl_2_ was added to a final concentration of 3 mM. *Pa*PepA was added at a final concentration of 100 nM in studies containing MnCl_2_, and to a final concentration of 1 μM in studies containing MgCl_2_ as the metal cofactor. Table 1 summarizes steady-state parameters described in Scheme 1 and obtained with different substrates.

**Table 1:** Steady-State Kinetic Parameters for Different *Pa*PepA Substrates and the Binding Affinity for Metal Ions

**Substrate**	*k*_cat_ (s^–1^)	*K*_M_ (mM)	*k*_cat_/*K*_M_ (s^–1^mM^–1^)
**Leu-pNA**	0.254 ± 0.005	0.16 ± 0.01	1.637 ± 0.1
**Phe-pNA**	0.115 ± 0.008	0.26 ± 0.05	0.44 ± 0.08
**Met-pNA**	0.084 ± 0.003	0.15 ± 0.02	0.56 ± 0.08
**Pro-pNA**	0.081 ± 0.003	1.20 ± 0.08	0.067 ± 0.005
**Arg-pNA**	0.052 ± 0.002	0.12 ± 0.02	0.44 ± 0.07
**Lys-pNA**	0.039 ± 0.002	0.28 ± 0.04	0.14 ± 0.02
**Ala-pNA**	0.037 ± 0.001	0.22 ± 0.03	0.17 ± 0.02
**Ile-pNA**	0.004 ± 0.000	0.10 ± 0.03	0.04 ± 0.01
**Val-pNA**	0.002 ± 0.001	0.05 ± 0.04	0.04 ± 0.03
**Leu-AMC**	0.56 ± 0.01	0.016 ± 0.001	35.4 ± 3.1
**AVLQAVLQSGFRKKAVLQSGFRKK-NH**_**2**_	0.098	0.155	0.635

**Metal**	*K*_D_ (nM)		
Mn^2+^	13.6 ± 0.5		
Mg^2+^	2137 ± 326		

**With 2 mM Leu-pNA**	*k*_cat_ (s^–1^)	*K*_ACT_ (mM)	*k*_cat_*/K*_ACT_ (s^–1^mM^–1^)
Mg^2+^	0.051 ± 0.002	3.0 ± 0.7	0.017 ± 0.004
Mn^2+^	0.274 ± 0.004	0.10 ± 0.01	2.7 ± 0.2
Fe^2+^	0.012 ± 0.003	>10	<0.0001
Co^2+^	0.137 ± 0.002	0.17 ± 0.01	0.80 ± 0.06
Ni^2+^	0.077 ± 0.003	2.5 ± 0.4	0.032 ± 0.005
Zn^2+^	0.009 ± 0.001	0.02 ± 0.01	0.5 ± 0.2

For assays investigating bicarbonate dependence, all buffers and stock solutions were extensively degassed prior to use.^25^ [MnCl_2_] was fixed at 3 mM and [Leu-pNA] at 2 mM, while the concentration of sodium bicarbonate was varied from 0 to 5 mM.

### pH Rate Profiles

A mixed buffer containing equimolar concentrations of HEPES, MES, and CHES was used. The buffer covered a full pH range from pH 5–10 in increments of 0.5 pH units.

Prior to determination of pH rate profiles, stability tests were performed. *PaPepA* was diluted to 10 μM in H_2_O or a mixed buffer at pH 6.5 or 8.5. HEPES, MES, and CHES were at 100 mM for the incubation period. *PaPepA* was then diluted to a final concentration of 100 nM in a reaction mixture at pH 8.0 (100 mM HEPES and 50 μM KCl). MnCl_2_ and Leu-pNA were present at saturating concentrations to assess whether *PaPepA* retained activity.

For pH profiles, each assay well contained a final concentration of 100 mM of each buffer component and 50 mM KCl. Other components (Leu, pNA, and MnCl_2_ or MgCl_2_) were varied. The *Pa*PepA enzyme was added at a final concentration of 100 nM when Leu-pNA or MnCl_2_ was varied, or 1 μM when MgCl_2_ was varied.

pH rate profiles were fitted to the following equation, where *y* is log (kinetic parameter), either *k*_cat_ or *k*_cat_/*K*_M_. *C* is the pH independent value of *y*.^26^
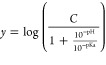
1

### Solvent Kinetic Isotope Effects (SKIEs) and Proton Inventories

**Scheme 2:**
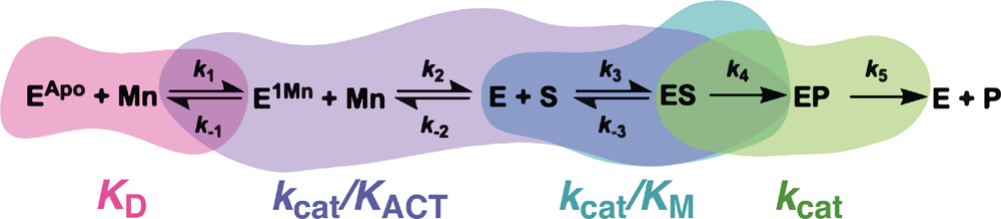
Reaction Scheme for the Steps in the Reaction Catalyzed by *Pa*PepA

SKIEs were determined by plotting saturation curves in H_2_O or 80% D_2_O. Viscosity studies (see below) showed no viscosity effects, and therefore, no correction was made to observed SKIEs to account for differences in the viscosity of H_2_O and D_2_O. Differences between reaction rates under initial velocity conditions were measured varying the concentration of either Leu-pNA or Mn^2+^, while keeping the other component constant and in excess (at least 5× the value of *K*_M_ or *K*_ACT_, Scheme 2). Experiments were performed at pH 8.0 (100 mM HEPES, with 50 mM KCl). For proton inventory studies, D_2_O was varied from 0 to 80% in 10% increments. All proton inventory studies were carried out on the same day by using the same enzyme stock.

When calculating SKIEs, *k*_cat_ and *K*_M_ were obtained from the Michaelis–Menten curves in H_2_O. The 80% D_2_O Michaelis–Menten curve was fit to the following equation:
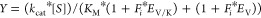
2where *F_i_* is the fraction D_2_O, and *E*_V/K_ and *E*_V_ are the isotope effects −1 on *k*_cat_/*K*_M_ and *k*_cat_, respectively.

Proton inventory data were fitted using the following modified Gross–Butler equation accounting for two transition state protons, where φ_1_ = φ_2_:
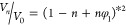
3where *V*_*n*_ is the kinetic parameter in D_2_O *V*_0_ is the kinetic parameter in H_2_O. Different models were fitted, and equations and values obtained are shown in Table S9.

### Viscosity Studies

Viscosity effects were studied by using sucrose as a viscogen. Relative viscosities for different concentrations of sucrose were taken from Bazelyansky et al.^27^ Concentrations of viscogen were as follows: 14% sucrose, η_rel_ = 1.5; 24% sucrose, η_rel_ = 2.2; and 32% sucrose, η_rel_ = 2.9.

Viscosity effects were fit to the following equation, where kinetic parameter_0_ and kinetic parameter_η_ are *k*_cat_ or *k*_cat_/*K*_M_ in the presence and absence of viscogen, respectively, η_rel_ is the relative viscosity of the solution, and *m* is the slope.
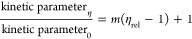
4

For *Pa*PepA saturation kinetics in PEG-8000, initial rates were measured at 25 °C at saturating concentrations of either Leu-pNA or MnCl_2_ while varying concentrations of the other reaction component, in the presence of 0 and 5% PEG- 8000 (*w/v*). All measurements were performed in triplicate.

### Qualitative Peptide Cleavage Assay

To further investigate *Pa*PepA’s substrate selectivity, we used a coupled assay to evaluate peptide cleavage. Reactions were carried out in 100 mM HEPES (pH 8.0) with 50 mM KCl. Two units of horseradish peroxidase (HRP) were added, alongside 3 mM MnCl_2_, 0.5 mM peptide, 100 nM *Pa*PepA, and 2 mg/mL l-amino acid oxidase (LAAO, Sigma). With successful peptide cleavage, oxidized TMB appears as a blue product within 10 min. If no cleavage occurs, the solution remains colorless. Formation of the blue color was monitored by examining the increase in absorbance at 370 nm over time. Assays were performed in a BMG Labtech POLARstar Omega plate reader, as described above.

### Alcohol Dehydrogenase Peptide Degradation Assay

A vial of MassPREP ADH digestion standard (Waters) was reconstituted in 200 μL of LC-MS grade water. Reaction mixtures were prepared by combining 25 μL of ADH solution, 3 mM MnCl_2_, and 100 nM *Pa*PepA (final concentrations) in a total volume of 50 μL buffer (100 mM HEPES pH 8.0, 50 mM KCl). For control reactions, the addition of *Pa*PepA was omitted. Samples and controls were each prepared in triplicate and incubated overnight, and reactions quenched with the addition of 50 mL of 10% TCA. Following the addition of acid, *Pa*PepA was added to control samples to a final concentration of 50 nM (equivalent to a concentration of enzyme in the reaction samples following acid quenching). Samples were concentrated to 20 μL. A 1 μL portion of this was diluted in 100 μL of LC-MS grade water. 10 μL was injected and analyzed on a SCIEX TripleTOF 5600+ mass spectrometer with an Eksigent 425 nanoLC configured in trap elute configuration. Each sample was loaded onto the trap (Waters ACQUITY M-Class symmetry column 180 μm × 20 mm) and washed with 0.05% TFA in water for 5 min at 5 μL/min. The trap was then switched in line with the Waters M-Class HSS1 analytical column, 75 μm × 150 mm, with a flow rate of 300 nL/min. Samples were eluted over 30 min using a linear gradient from 98% A (100% water 0.1% FA) 2% B (80% MeCN, 100% water 0.1% FA) to 60% A, 40% B linearly over 12 min, rising to 5% A, 95% B, before returning to 98% A to re-equilibrate. Eluate from the column was sprayed directly into the nanospray source of the mass spectrometer. MS data were collected from 400 to 1250 *m*/*z* as the survey scan, and under data-dependent acquisition (DDA) setting, the top 20 strongest peptides were selected for MS/MS using CID fragmentation. Peptide mass tolerance was 20 ppm on the MS and 0.1 Da on the MS/MS.

Identification and analysis of ADH peptides were performed using the Skyline open-access software package (version 22.2). Raw data were extracted using msConvert (ProteoWizard) and the data searched using Skyline against the ADH1 sequence from *Saccharomyces cerevisiae* (UniProt: P00330). The digestion enzyme was set as trypsin, allowing up to three missed cleavages. Searches encompassed peptides between 400 and 1250 *m*/*z* with a mass tolerance of 0.3 *m*/*z*. Carbamidomethyl was set as a fixed modification for cysteine residues. Data were searched for the protonated 1+, 2+, and 3+ charge states of each peptide. Table S3 lists all of the peptides identified.

Peaks were integrated, and areas were used to qualitatively assess differences in peptide abundances across *Pa*PepA-digested samples and controls. Peptides present in control samples but absent in reactions (integrated area <1000) were classified as “digested”.

### AVLQSGFRKK-NH_2_ Degradation Time Course

Time-course reaction mixtures to investigate the breakdown of AVLQSGFRKK-NH_2_ were prepared in duplicate to a final volume of 500 μL. Conditions were as follows: 100 mM HEPES at pH 8.0, 50 mM KCl, 3 mM MnCl_2_, 1 μM *Pa*PepA WT, and AVLQSGFRKK-NH_2_ at 25, 50, 100, or 200 μM. 50 μL was removed from the reaction mixture at time points 30 s, 1 min, 3 min, 5 min, 15 min, 30 min, 60 min, 3 h, 6 h and added to an equal volume of 10% TCA to quench. Control samples were 25, 50, 100, or 200 μM AVLQSGFRKK-NH_2_ incubated in buffer for the full 6 h, or 25, 50, 100, or 200 μM AVLQSGFRKK-NH_2_ incubated in the presence of 1 μM inactive mutant PepA (D348A).

Samples were centrifuged at 14k rpm for 10 min, and 10 μL of each run was on a Waters H-Class HPLC and ACQUITY QDa Mass Detector (HPLC gradient, masses monitored, and the QDa method for acquisition detailed in Tables S4 and S5). TargetLynx (Waters) was used to detect and integrate peptide peaks. For each peptide intermediate, [M+H^+^] and [M+Na^+^] adducts were searched in a single-ion recording (SIR) method. Table S6 lists the fitted data for AVLQSGFRKK-NH_2_ decay.

### Electron Paramagnetic Resonance (EPR) Spectroscopy

EPR spectra were obtained with a Bruker EMX 10/12 spectrometer running Xenon software and equipped with an ELEXSYS Super Hi-Q resonator at an operating frequency of ∼9.5 GHz with a 100 kHz modulation. EPR spectra for Mn titrations were recorded at room temperature using a 160 mT field sweep centered at 350 mT, a time constant of 40.96 ms, a conversion time of 133.53 ms, and a 480-point resolution. An attenuation of 10 dB (20.7 mW power) and a modulation amplitude of 1 mT were used. Spectra were phase- and background-corrected, and the double integral was obtained using the Xenon software.

Low-temperature EPR spectra were obtained at 120 K with an ER4141 VTM Nitrogen VT unit (Bruker) using a 300 mT field sweep centered at 315 mT, a time constant of 40.96 ms, a conversion time of 17.79 ms, and a 6000-point resolution. An attenuation of 10 dB (20.7 mW power) and a modulation amplitude of 0.5 mT were used.

For acquiring EPR spectra, 20 μM PaPepA was used in 100 mM HEPES pH 7.5, 50 mM KCl with increasing concentration of MnCl_2_: 0–30 μM in steps of 2, 35, and 40 μM (18 steps). For the calibration curve, the same buffer and steps were employed in the absence of enzyme.

### Isothermal Titration Calorimetry

Purified *Pa*PepA (100 μM) was buffer exchanged into ITC buffer (50 mM Tris, 140 mM NaCl, pH 7.4) using a Millipore Amicon Ultra-4 centrifugal unit (10 kDa MWCO). The protein was syringe-filtered through a 0.2 μM filter and diluted to 50 μM. 3 mL solutions of filtered metal were also prepared. A 400 μM solution of MnCl_2_ and a 1 mM solution of MgCl_2_ were prepared by dilution of 1 M metal chloride stocks (Sigma) into ITC buffer.

Buffer, protein, and metal solutions were degassed. Following extensive washes with ITC buffer, the protein was loaded into the cell (volume 1.43 mL) of a VP-ITC (MicroCal) and MnCl_2_ or MgCl_2_ solution was loaded into the syringe (total volume 300 μL). Experiments were carried out using the following settings: temperature = 25 °C, duration = 4 s, spacing = 210 s, filter period = 2 s, for 35 injections (one injection of 2 μL followed by 34 injections of 8 μL). Data were analyzed using Origin(Pro), Version 2021 (OriginLab Corporation, Northampton, Massachusetts, USA), and MicroCal PEAQ-ITC analysis software (Malvern). Table S7 lists all of the fitted and calculated parameters.

### Pre-steady-State Kinetics

An Applied Photophysics SX20 stopped-flow spectrofluorimeter was used to perform kinetic measurements under pre-steady-state conditions. The instrument was equipped with a xenon lamp, a 5 μL mixing cell, and a circulating water bath to maintain constant temperature. All reactions were carried out in 100 mM HEPES (pH 8.0) and 50 mM KCl at 25 °C. Enzyme and metal ions were kept in one syringe, while the other substrate (Leu-pNA or Leu-AMC) was kept in the second syringe. The reaction was triggered by rapid mixing of 55 μL from each syringe. Three traces with 10,000 points per trace were collected per experiment.

For absorbance measurements, product formation was monitored by reading the pNA absorbance at 405 nm (5 mm path length). Multiple turnover rates were measured in the presence of saturating concentrations of both metal and Leu-pNA. *Pa*PepA was present at 1 or 10 μM, MnCl_2_ was present at 3 mM, and Leu-pNA was present at 250 μM. For fluorescence measurements, a path length of 1 mm was used. Reactions were followed by monitoring the appearance of a 7-amino-4-methylcoumarin (AMC) fluorophore following *Pa*PepA-catalyzed breakdown of Leu-AMC. An excitation wavelength of 341 nm was used with a 400 nm cutoff filter for the emission. Slits were 1 nm for excitation and 2 nm for emission.

Experiments under single turnover conditions monitored fluorescence change with a detector voltage of 300 V, 0.1 μM Leu-AMC, 1.5 mM MnCl_2_, and varying *Pa*PepA (0.5, 1, 2, 4, 8, 16, or 32 μM). Experiments under multiple turnover conditions monitored fluorescence change with a detector voltage of 250 V, 50 μM Leu-AMC, 1.2 μM *Pa*PepA and varying concentrations of MnCl_2_ (0.2, 0.4, 0.6, 1.2, 2.4, 6, 9, 12, 18, 24, 60, 120, 1200, or 3600 μM).

Calibration curves of known concentrations of AMC were performed at detector voltages of 250 and 300 V. STO and MTO curves were divided by the slope of these calibration curves to convert the change in fluorescence observed to the concentration of AMC produced.

### Fitting of Kinetic Data Using KinTek Global Explorer

Pre-steady-state data were imported into KinTek Global Explorer and fit to linear or single-exponential equations using the aFit function to obtain standard deviation estimates. A kinetic model with five steps was considered, and rate constants and outputs were fitted according to this model. Initial estimates for rate constants were based on *k*_cat_ and *K*_M_ values. The FitSpace Editor was employed to determine confidence intervals for each of the rate constants. More details including model fitted and parameters used are available in the Supporting Information. Table 2 lists global fits results.

**Table 2:** Global Fit of Multiple and Single Turnover Data Shown in Figure 4

	**Best-fitted value**	**Lower boundary**	**Upper boundary**
*k*1 (μM^–1^ s^–1^)	1000 ± 53	35.3	2540
*k*2 (μM^–1^ s^–1^)	4.9 ± 0.3	9.6	1000
*k*-2 (s^–1^)	450 ± 26	880	>2000
*k*3 (μM^–1^ s^–1^)	2.3 ± 0.1	1.3	2.5
*k*-3 (s^–1^)	47.9 ± 0.6	24.8	52.8
*k*4 (s^–1^)	6.3 ± 0.1	6.1	6.5
*k*5 (s^–1^)	13.4 ± 0.1	12.5	14.8

### N-Terminal-Sequence-Pattern-Finder Script

To identify sequence patterns in the N-termini of different proteins in the genome of *P. aeruginosa*, a Perl script (multiplatform) that traverses an input file having protein sequences as FASTA format (Project “N-terminal-sequence-pattern-finder” on GitHub), that employs pattern matching on strings generating a list of proteins as output that begin with a certain sequence, was used. In our case, we searched for proteins starting with string “ML”, with the assumption that most N-terminal methionine residues are or can be removed posttranslationally.

## Results and Discussion

### *Pa*PepA Exhibits a Broad and Unexpected Substrate Selectivity

**Figure 1:**
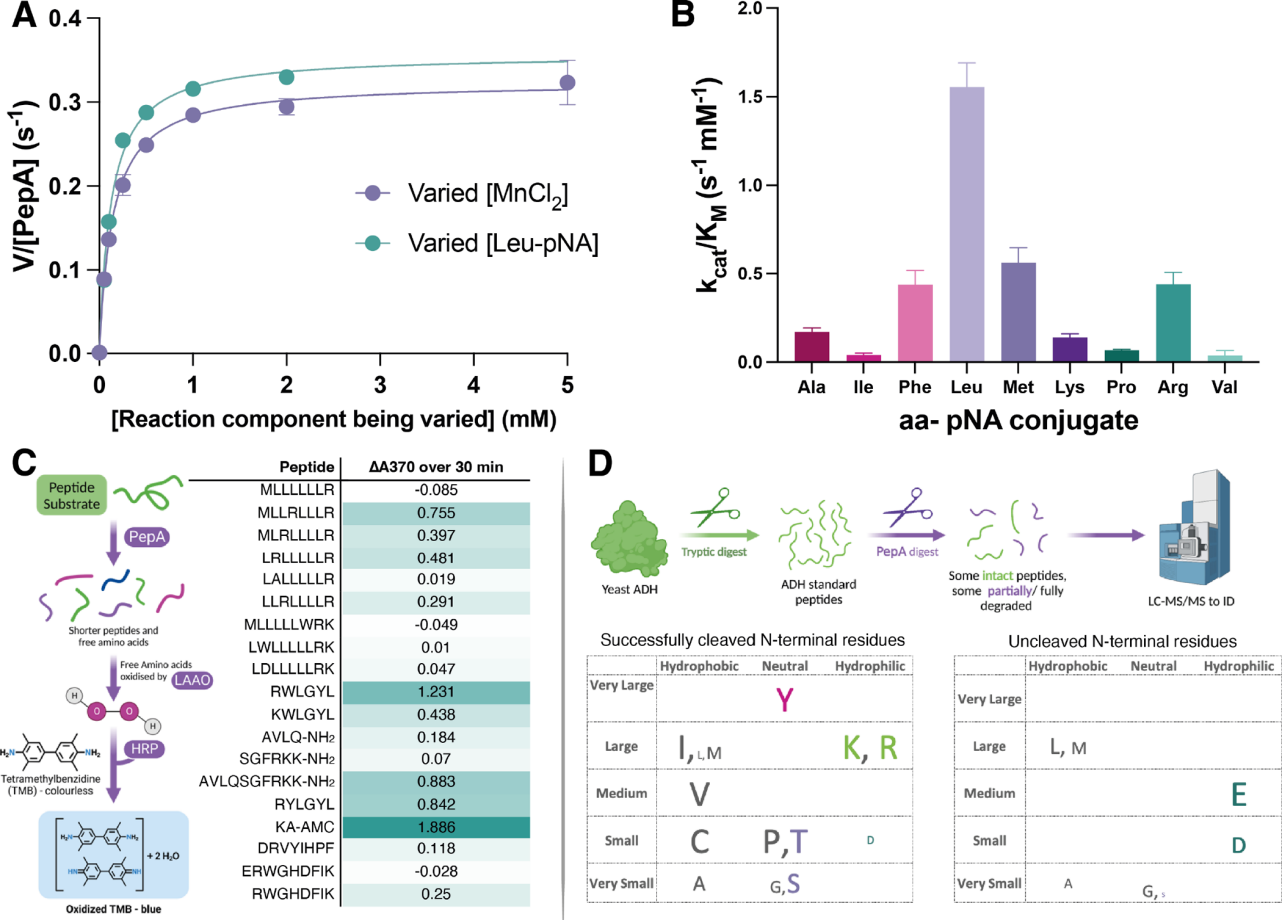
Peptide and aa-pNA conjugates are cleaved by *Pa*PepA. (A) Initial velocity plotted as a function of substrate concentration, fit to a Michaelis–Menten curve. (B) *k*_cat_/*K*_M_ values for various amino acids conjugated to pNA. (C) Assay schematic representing a coupled colorimetric assay used to evaluate peptide cleavage for hydrophobic peptides, which were impossible to assess using mass spectrometry, and table showing the change in absorbance at 370 nm after 30 min of incubation with *Pa*PepA. (D) Top: scheme for *Pa*PepA digestion of ADH peptides; bottom: plots evaluating the likelihood of *Pa*PepA to cleave different N-terminal residues. Left: *Pa*PepA cleaved N-terminal residues and their properties. Right: Uncleaved N-terminal residues. Font size corresponds to the percentage of peptides with a specific N-terminal residue that were not cleaved by the enzyme. Key: pink, aromatic; gray, nonpolar; teal, negatively charged; green, positively charged; purple, polar. Data on (B) and (C) data are shown as mean ± SEM from three replicates.

*Pa*PepA was evaluated for its aminopeptidase activity against a variety of amino acids conjugated to pNA (aa-pNA). aa-pNA conjugates act as substrate mimics for peptides, combining an amino acid at the N-terminus with a *p*-nitroaniline leaving group on the C-terminus (Scheme 1, middle). Table 1 summarizes steady-state parameters described in Scheme 1 and obtained with different substrates. All pNA conjugates tested were cleaved to some extent (Figure 1B), but *Pa*PepA demonstrated greater catalytic efficiency with leucine, methionine, phenylalanine, and arginine-pNA. Variation across *k*_cat_/*K*_M_ for each aa-pNA appears to be primarily driven by differences in *k*_cat_. *K*_M_ shows far less variation across aa-pNA conjugates, except for Pro-pNA. When *Pa*PepA was tested against Leu-AMC, *K*_M_ was reduced in comparison with Leu-pNA (Table 1). In terms of the amino acid series tested as pNA conjugates, our findings largely reflect the preferences of *Pa*PepA homologs from the M17 aminopeptidase family. Tomato leucine aminopeptidase (LAP) preferentially cleaves N-terminal methionine, arginine, isoleucine, leucine, or valine.^28^ PepA from *E. coli* favors N-terminal alanine, phenylalanine, and arginine residues.^28^ PepA from *Pseudomonas putida* (*Pp*LAP) preferentially cleaves substrates containing terminal bulky hydrophobic residues, such as leucine, isoleucine, and phenylalanine, and shows poor cleavage of small or negatively charged N-terminal glycine, valine, aspartate, and glutamate residues.^29^ To date, aspartate and glycine at the amino terminal position tend to be the worst substrates for all LAPs that have been studied.^28^ The residue at the penultimate (P1′) position also strongly influences cleavage, with proline, aspartate, glycine, and lysine at P1′ reducing *k*_cat_ for M17 LAPs.^30^ In general, for microbial PepA homologs, a wider range of P1′ residues can be tolerated if P1 is a leucine or arginine.^31^ The residue at the P2′ position has also been shown to affect *k*_cat_ values of Arg-Gly-Xaa (where Xaa is any amino acid) tripeptides, although this varies substantially across M17 LAPs from different organisms.^7,30^ Arginine residues at P2′ tend to slow *k*_cat_,^8^ whereas the impact of a P2′ aspartate residue is variable, enhancing peptide cleavage by *E. coli* PepA, but not by plant LAPs.

Analyzing sequences from the *P. aeruginosa* proteome that contain methionine and leucine in their N-termini revealed that protein sequences with N-terminal “ML” frequently possessed an N-terminal sequence rich in subsequent leucine residues, usually followed by arginine residues (Figures S2 and S3), where 339 proteins have a sequence starting with “ML”, out of a total of 5893 proteins from *P. aeruginosa* PA14. Therefore, a series of eight-to-nine-residue M/L-rich peptides was designed and tested as substrates. A coupled assay (Figure 1C) was used to evaluate the *Pa*PepA-mediated cleavage of these peptides. Mass spectrometry was unsuitable as certain peptides tested—particularly sequences that were rich in leucine—were not amenable to LC-MS analysis due to their high hydrophobicity. During the assay, peptide breakdown by *Pa*PepA results in the release of free amino acids. Amino acids are oxidized by l-amino acid oxidase (LAAO) to form hydrogen peroxide. HRP reduces hydrogen peroxide, which results in oxidation of 3,3′,5,5′-tetramethylbenzidine (TMB). Oxidized TMB appeared as a blue product. Hence, incubation of *Pa*PepA with LAAO, HRP, TMB, and a viable peptide substrate results in a color change of the reaction solution to blue, which can be detected at 370 nm. If no cleavage occurs, the solution remains colorless. To assess whether various peptides could act as *Pa*PepA substrates, reaction mixtures were incubated for 20 min in the presence or absence of *Pa*PepA. If a threshold change from the start to the end of the experiment was observed over 0.1 absorbance units, and this change was not observed in the absence of added *Pa*PepA, this was classified as successful cleavage (Figure 1C).

These experiments revealed that an arginine was required within the first four residues of these hydrophobic peptides for successful cleavage. The presence of tryptophan or aspartate residues at the P1′ position in the M/L-rich peptides was not tolerated. LALLLLLR was the only tested peptide with a stretch of four nonpolar residues at its N-terminus that was successfully cleaved; all other cleaved peptides required at least one polar or charged residue within the first four positions.

Subsequently, various peptides containing charged residues and diverse sequences were tested, revealing that tryptophan residues were accepted if they were adjacent to a P1 lysine or arginine. Surprisingly, incubation of PepA with peptides containing AVLQ motifs at their N-terminus resulted in the formation of a strong blue color, although assays with Ala-pNA and Val-pNA (Figure 1B) had suggested that N-terminal alanine and valine residues were poor substrates.

To further investigate the substrate selectivity of *Pa*PepA using peptides containing high sequence variation, MassPREP standard peptides from the tryptic digest of yeast ADH were incubated in the presence or absence of *Pa*PepA (Figure 1D). Table S3 lists all of the peptides represented in the MassPREP digest, encompassing diverse sequences. Such experiments revealed unexpected substrate selectivity for *Pa*PepA (Figure 1D, Figure S3). Aa-pNA assays (Figure 1B) implied that methionine, leucine, phenylalanine, and arginine (M/L/R/F)-rich substrates would be strongly preferred as these residues exhibit the greatest catalytic efficiency of all tested pNA conjugates, while peptides with N-terminal alanine, isoleucine, lysine, and valine would be very poor substrates. However, peptides with N-terminal alanine, isoleucine, lysine, and valine were successfully cleaved.

**Figure 2:**
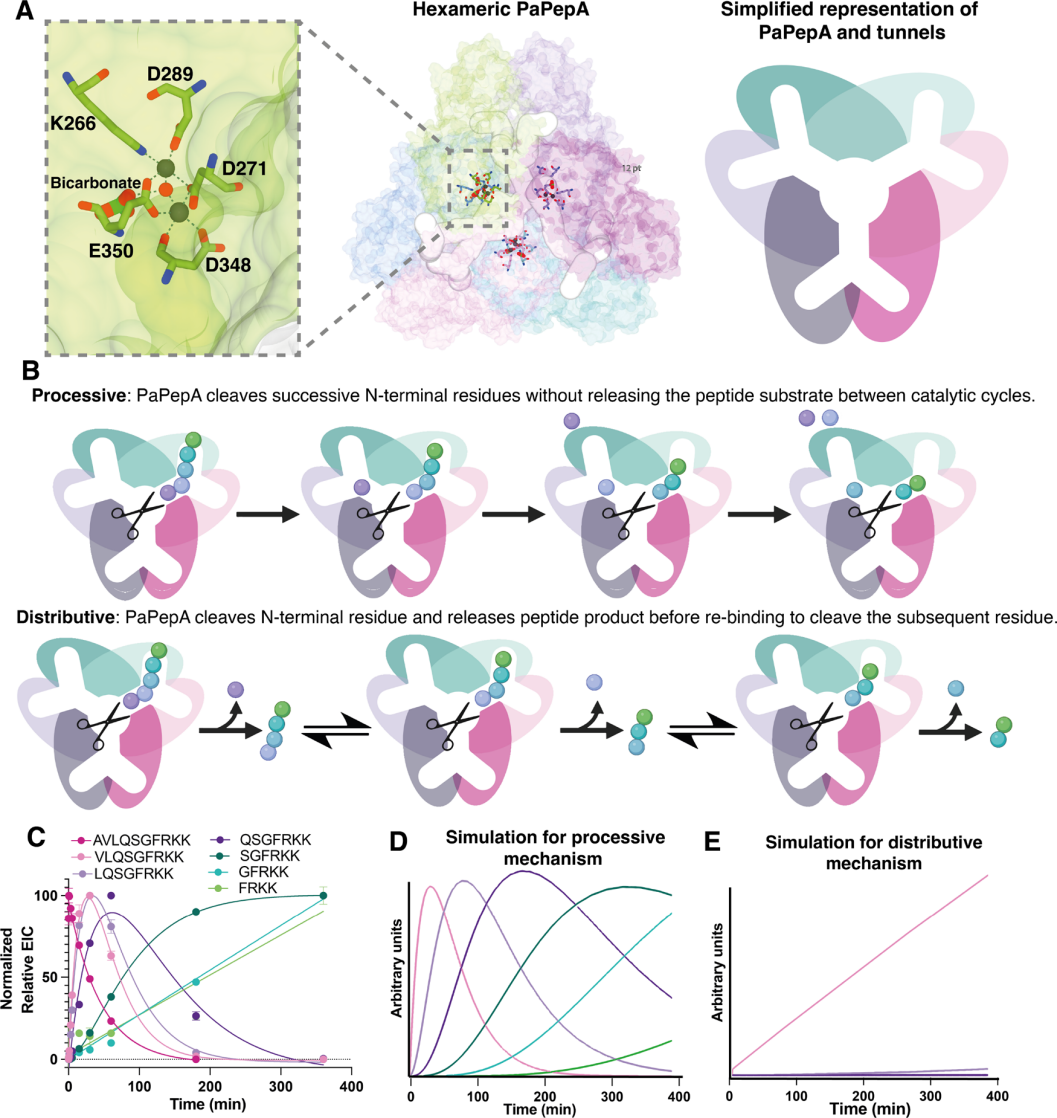
(A) *Pa*PepA active sites (active site residues are displayed as sticks; metals are displayed as gray spheres) and CAVER^34^-predicted tunnels through which peptide substrates can enter (displayed in white). Each subunit of the hexamer is colored differently. The inset depicts an active site from a single monomer in relation to tunnel location. (Right): simplified diagram of the *Pa*PepA hexamer. (B) Scheme showing the difference between processive and distributive modes of peptidase action. (C) Time-course assay investigating peptide hydrolysis using LC-MS to detect peptide fragments of reaction with 25 μM peptide (AVLQSGFRKK-NH_2_) and 1 μM *Pa*PepA quenched at different time points. Each data point was measured in duplicate, and the data were fit to single (AVLQSGFRKK-NH_2_), or double (VLQSGFRKK-NH_2_, LQSGFRKK-NH_2_, QSGFRKK-NH_2,_ SGFRKK-NH_2_, GFRKK-NH_2_, FRKK-NH_2_, and RKK-NH_2_), exponential equations using GraphPad Prism. Averages are shown. (D) Simulation using rates obtained during exponential fitting of data in (C) in a processive mechanism. (E) Simulation using rates obtained during exponential fitting of data in (C) in a distributive mechanism. Rate constants for peptide binding and dissociation were set at 100 μM^–1^ s^–1^ and 10 s^–1^, respectively. For clarity, one site is depicted to be turning over as opposed to all active sites working in unison. Equations and additional details available in Supporting Information.

Time-course experiments investigating the degradation of a long peptide substrate were conducted to determine whether cleavage occurred in a processive or distributive manner (Figure 2). If the cleavage process were processive, then *Pa*PepA would bind to peptides and catalyze the hydrolysis of multiple amino acid residues before releasing truncated peptide products (Figure 2B, top). Conversely, if the reaction followed a distributive mechanism, *Pa*PepA would bind to a peptide substrate, remove the N-terminal amino acid, release a peptide intermediate, and require rebinding for the removal of subsequent amino acids (Figure 2B, bottom).

Preliminary assays indicated that peptide AVLQSGFRKK-NH_2_ was cleaved sequentially to a small tripeptide fragment by *Pa*PepA (Figure S4). This peptide was therefore employed for progress curves with varying substrate concentrations.^32^ The assay was quenched at different time points and analyzed by LC-MS, showing the cleavage of the parent peptide into shorter products. The formation and disappearance of these products over time were analyzed by selected ion recording (SIR) and extracted ion chromatograms (EIC). Assessing the pattern and rates of formation and consumption of each intermediate implies that *Pa*PepA can use the peptide products as substrates after cleaving the N-terminal (P1) residue, functioning processively^33^ for the first several N-terminal residues, as truncated peptide intermediates do not accumulate in early time points (Figure 2C). Fitting data to exponential equations to obtain rates of formation and decay of peptide intermediates and then using the fitted values to generate progress curve simulations in Kintek Global Explorer further corroborate a processive model up until the formation of a tetrapeptide (FRKK), as empirical data for formation and decomposition of intermediates show no accumulation of parent peptide −1 amino acid (VLQSGFRKK-NH_2_). Accumulation would be expected for a distributive mechanism as the parent peptide is in large excess in comparison to any intermediates (Figure 2D,E). Details of simulated models as well as all data showing progress curves at different peptide concentrations are available in Supporting Information Note 1.

Assessing progress curves for the degradation of the parent peptide (Figures S5 and S6) and using a calibration curve to convert mass peak area into concentration enables data fitting in Kintek Global Explore to obtain kinetic parameters *k*_cat_, *K*_M_, and *k*_cat_/*K*_M_.^32^ This reveals similar *K*_M_ values for the AVLQSGFRKK-NH_2_ peptide and Leu-pNA, and that *k*_cat_ for cleavage of this peptide substrate is much lower than that observed for Leu-pNA or Leu-AMC. This is potentially due to the processive nature of *Pa*PepA. Instead of binding the substrate and releasing the product, the enzyme undergoes a more complex kinetic cycle in which the product may then act as a substrate itself (Figure 2B).

These results suggest that peptide substrate acceptance by *Pa*PepA is a complex and nuanced process. Successful cleavage of a residue conjugated to pNA is not necessarily translated into the capacity to catalyze peptide hydrolysis. The identity of the full peptide, rather than the P1 residue alone, defines whether or not a peptide may act as a *Pa*PepA substrate. This demonstrates a complex *Pa*PepA substrate selection landscape.

### Product Inhibition Studies

Since *Pa*PepA generates free amino acids when peptides are hydrolyzed, we hypothesized that it could be inhibited by amino acid products. When evaluating leucine, methionine, and phenylalanine as inhibitors, we obtained IC50 values of 51 μM, 2.5 mM, and 15 mM, respectively (Figure S7A). Leucine inhibition was further evaluated, displaying competitive inhibition in relation to Leu-pNA and *K*_i_ = 601 μM (Figure S7B). Estimates indicate that in *E. coli*, the intracellular concentration of leucine in minimal media is 1.7 mM, with a steady-state level of 5.3 mM. This is higher than the inhibition constant,^35,36^ indicating that *Pa*PepA can be inhibited when leucine—and potentially other—free amino acids are abundant.

### *Pa*PepA Operates across a Narrow pH Range

Kinetic parameters (turnover number *k*_cat_*;* the catalytic efficiency *k*_cat_/*K*_M_ for the Leu-pNA substrate, and *k*_cat_/*K*_ACT_ for Mn^2+^) for the *Pa*PepA-mediated cleavage of Leu-pNA were evaluated in the pH range 6.5–8.5 (Figure S8). Enzyme stability was determined to span this range by preincubating *Pa*PepA at extreme pH values for 1 h and then diluting and performing a standard reaction at pH 8.0 (Figure S8A). *k*_cat_/*K*_ACT_ describes the activation efficiency, where *K*_ACT_ is the concentration of metal required to reach half maximal activation. A mathematical definition of these kinetic parameters is available in the Supporting Information (Supporting Information, Note 3), highlighting the kinetic complexity of *k*_cat_/*K*_ACT_, how *K*_ACT_ differs from *K*_D_, and its dependence on substrate concentration and chemical steps.

Below pH 6.5, *Pa*PepA exhibited no activity, and above pH 8.5, metal salts precipitated from solution in the mixed buffer. Prior work on homologous M17 LAPs has also shown aminopeptidase inactivity below pH 6.5, and pH optima from 8.0 to 9.0 for this class of enzymes.^37−40^ Regardless of which substrate concentration was being varied, *k*_cat_, *k*_cat_/*K*_M_, and *k*_cat_/*K*_ACT_ increased with increasing pH. Data fitting to eq 1 yielded an apparent p*K*_a_ for *k*_cat_ of 7.9 ± 0.1, and for *k*_cat_/*K*_M_ while Leu-pNA was varied of 7.6 ± 0.1. The *k*_cat_/*K*_ACT_ curve obtained for MnCl_2_ displayed a substantially higher p*K*_a_ value at 12.2 and lacked an obvious plateau. The errors associated with this curve are larger than expected (Figure S8B) likely due to the lack of a defined plateau; therefore, we did not consider this in mechanistic discussions. Subsequent assays were all carried out at pH 8.0 in the plateau region to limit the effect of pH variation on rates and to prevent issues with metal precipitation at higher pH.

Taken together, these results and their fit to eq 1 suggest that one group must be deprotonated for substrate or metal binding and catalysis. As the p*K*_a_ values are the same (within error) for *k*_cat_/*K*_M_ and *k*_cat_ for Leu-pNA, two possibilities arise: (1) p*K*_a_ values are reflecting the same group, which has to be deprotonated in steps contributing to *k*_cat_/*K*_M_ and *k*_cat_, (2) p*K*_a_ values are reflecting two distinct groups with similar apparent p*K*_a_ values, which have to be deprotonated in steps contributing to *k*_cat_/*K*_M_ and *k*_cat_. Scheme 2 summarizes the steps contributing to each kinetic parameter. Although the precise nature of this group is undetermined, *Pa*PepA’s structure (discussed below) revealed a bicarbonate ion bound closely to the active site. This was also observed on other PepA orthologs (*Ec*PepA, *Pp*PepA, and bovine lens peptidase^41^) and previously proposed to act as a general base in the mechanism.^31^ The previously proposed mechanism for *Ec*PepA (Figure S9A) contemplates the first possibility highlighted above, in which bicarbonate acts as a catalytic base to activate metal-bound water, and also takes part as carbonic acid in proton shuttle enabling dissolution of the tetrahedral intermediate.^25^ However, these two distinct roles would require pH rate profiles depicting an unprotonated group for steps included on *k*_cat_/*K*_M_, but a protonated group for steps included on *k*_cat_, which is not observed here. Furthermore, we have not observed activation or increase in activity after bicarbonate was removed and reintroduced to reactions (Figure S9C), and a mutation in EcPepA^25^ that abolishes crucial interactions between an arginine residue and bicarbonate did not result in decreased in activity.

**Scheme 3:**
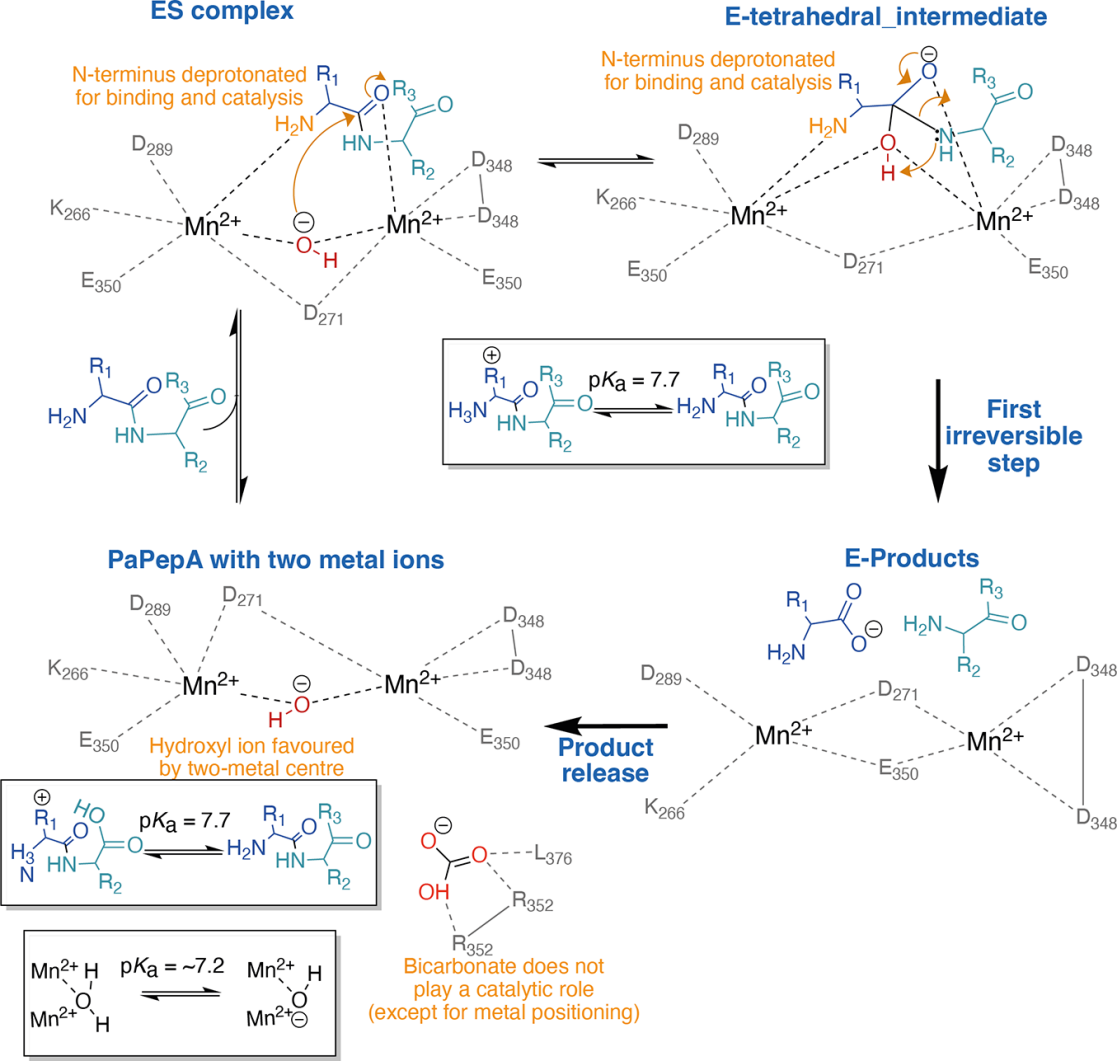
Catalytic Mechanism Proposal for PaPepA Mechanism proposed here, in which the metal coordinated water can give rise to the p*K*a observed for *k*_cat_/*K*_M_, while deprotonated peptide N-terminus could contribute to the *k*_cat_ profile. On the right of both mechanisms, there is a scheme of expected pH rate profiles for each mechanism.

Considering a group that needs to be deprotonated for steps included on *k*_cat_, two possibilities are put forward as likely mechanistic scenarios, both not involving bicarbonate actively in acid–base catalysis. Manganese-dependent enzymes with binuclear binding sites, such as *Pa*PepA, coordinate a water molecule between the two manganese atoms. This has been shown to significantly decrease the p*K*_a_ for the metal-bound water, bringing it close to 7.2–7.5 in arginase.^42,43^ We therefore propose an alternative catalytic mechanism (Scheme 3), in which the dinuclear manganese system acts as a Lewis acid, binding a water molecule simultaneously to two Mn^2+^ and leading to OH^–^ formation. This is followed by OH^–^ nucleophilic attack of the peptide bond to be broken and then collapse of the tetrahedral intermediate and peptide cleavage. Additionally, based on a protein complex structure bound to the inhibitor bestatin (see “Structure of PaPepA” below), the N-terminus of the peptide is coordinated to one of the Mn^2+^ ions and therefore likely deprotonated. Metal coordination contributes to substrate positioning during catalysis. Both peptide N-terminal amine and water coordinated in the metal center have p*K*_a_ values between 7.0 and 8.0 and therefore either could be giving rise to the pH rate profiles seen on *k*_cat_/*K*_M_ and *k*_cat_.

### *Pa*PepA Can Use Several Divalent Metals for Catalysis

**Figure 3:**
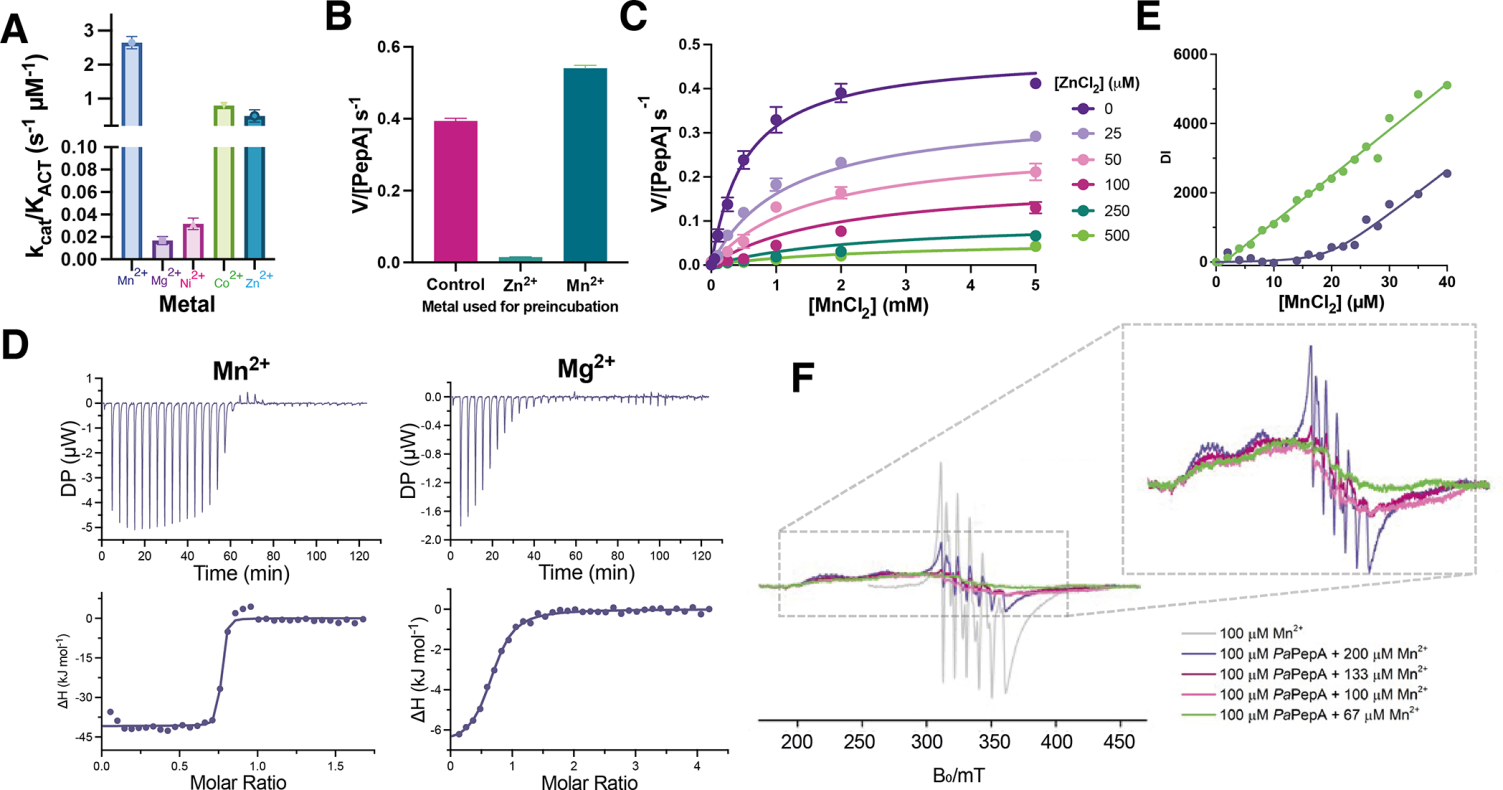
*Pa*PepA metal binding and catalysis. (A) *Pa*PepA activity with different divalent metals assessed through *k*_cat_/*K*_ACT_. Leu-pNA was present in all assays at a concentration of 2 mM. (B) The effects of Zn^2+^ and Mn^2+^ preincubation, followed by extensive dialysis in metal-free buffer and subsequent addition of excess Mn^2+^. (C) Michaelis–Menten plots showing inhibition of *Pa*PepA by Zn^2+^. Data were fit to a noncompetitive mixed model of inhibition. (D) Representative plots of the ITC data for titrations of MnCl_2_ (left) and MgCl_2_ (right) with *Pa*PepA. The top panel depicts raw data, and the bottom panel depicts the resulting enthalpy changes as a function of molar addition of metal as well as the best fit to a single binding site model. (E) Plot of room-temperature EPR double integrals against Mn^2+^ concentration for samples without enzyme (teal dots) and in the presence of 20 μM *Pa*PepA (purple dots). The calibration curve obtained from the samples without *Pa*PepA is fit to a linear model (green line) and data obtained from the samples containing *Pa*PepA with a fit assuming 0.9 equiv Mn^2+^ with a *K*_D_ of 174 nM (purple curve). (F) Frozen solution EPR spectra of *Pa*PepA in the presence of various ratios of Mn^2+^: enzyme, upper box zoomed-in data without the metal in buffer control. For panels (A), (B), and (C), data are mean ± SEM across three replicates. For Figure 3D, experiments were performed in two separate biological replicates, one shown here for illustration, and all data were acquired in Figure S11.

In other aminopeptidases, two metals are bound to the active site and metal selection varies. While some enzymes prefer Zn^2+^, others have a mixed metal biding site with Zn^2+^/Mn^2+^, in which Zn^2+^ is proposed to bind to a higher affinity site. No available studies have quantitatively evaluated the aminopeptidase metal binding and activation. *Pa*PepA did not copurify with Zn^2+^ if metal was absent from the purification buffer, according to crystal structures and fluorescence data (Figure S10). Zn^2+^ activated *Pa*PepA in the absence of other metals, although this was to a lower extent than Mn^2+^ (Figure 3A,B). The relatively high *k*_cat_/*K*_ACT_ for Zn^2+^ is driven by a low *K*_ACT_, while *k*_cat_ is very low (Table 1; for Zn^2+^, *K*_ACT_ is 20 μM and *k*_cat_ is 0.009 s^–1^, while for Mn^2+^, *K*_ACT_ is 100 μM and *k*_cat_ is 0.27 s^–1^).

The closest homologue to PA14 PepA, sharing a 79.6% sequence identity, is *P. putida* PpPepA. *Pp*PepA exhibits an optimal rate if the high-affinity site is occupied with Zn^2+^ and the second site harbors Mn^2+^.^29^ Previous work on M17 aminopeptidases has highlighted their ability to use a range of divalent metal cations for catalysis.^28,29^ Structures of PepA homologs showed two metal binding sites per monomer, one which tightly binds Zn^2+^ and another, more readily exchangeable site which may be occupied by Mg^2+^, Zn^2+^, Co^2+^, or Mn^2+^.^29^ Structural studies of PepA from *P. putida*, bovine lens, and *E. coli* suggest that Zn^2+^ is essential for PepA-mediated catalysis, as Zn^2+^ copurifies with PepA from each of these organisms and consistently appears in the high-affinity binding site in crystallographic studies.^29,44^ For most organisms, the low-affinity site is suggested to bind Mg^2+^ or Mn^2+^, as these cations are potent activators of LAPs from plants, animals, and microbes.^45^

Unexpectedly, a subset of studies suggests that despite the above structural findings and classification of *Ec*PepA as a “dizinc-dependent aminopeptidase”, Zn^2+^ acts as a potent inhibitor of this enzyme. It is instead proposed that *Ec*PepA is likely to use Mn^2+^ or Mg^2+^ for its catalytic function.^7,31^ In summary, metal selection and activation in related aminopeptidases are poorly understood.

To further understand metal occupancy in the tight-binding site, a cycle of metal preincubation followed by extensive dialysis was followed as previously conducted with PepA homologs.^29^ This aimed at occupying the high-affinity site with the first metal of choice, using dialysis to remove weakly bound metal from the low-affinity site and then adding a second metal to fill it. In contrast, when *Pa*PepA was preincubated with Zn^2+^, dialyzed, and then exposed to Mn^2+^, it displayed limited activity in comparison to *Pa*PepA which had been preincubated with Mn^2+^, or *Pa*PepA which had not undergone preincubation but which had been exposed to excess Mn^2+^ at the time of the assay (Figure 3B). If Zn^2+^ and Mn^2+^ were both added to the pNA assay mixture without preincubation, Zn^2+^ showed a strong inhibitory effect on *Pa*PepA (Figure 3C), with a *K*_i-Zn2+_ = 12.2 μM, lower than the *K*_ACT_ for Mn^2+^ of 100 μM. Data were fit to a noncompetitive model of inhibition, with α = 5.6. In this model, Zn^2+^ binds preferentially to free *Pa*PepA but is also capable of binding to the *Pa*PepA-substrate complex.^46^ The strong inhibitory effect observed highly resembles the initial findings on *E. coli* PepA^77^.

To assess which metal is most likely to act as the *Pa*PepA activator in vivo, it is important to consider the concentrations of various metals in the *P. aeruginosa* cytosol. Schalk and Cunrath and Cunrath et al. used inductively coupled plasma mass spectrometry (ICP-MS) to probe the intracellular concentrations of different metals in *P. aeruginosa* PA01 when grown under different media conditions.^47,48^ They found that cells grown in metal-restricted casamino acid medium possessed undetectable levels of Mn^2+^ in their cytosol. When cells were grown in LB, Mn^2+^ was present at 0.32 mM. Co^2+^ was not detected in *P. aeruginosa* in any of the conditions investigated (detection limit: 10 nM); therefore, this is highly unlikely to be the metal *Pa*PepA uses in vivo despite its moderate enzyme activation observed in Figure 3. Contrastingly, Mg^2+^ displayed intracellular concentrations of 100–500 mM depending on the growth media used.^48^ As the concentration of cellular Mg^2+^ is markedly higher than that of Mn^2+^, there is the potential that this outweighs the greater binding affinity of *Pa*PepA for Mn^2+^. Competition for metals is fierce during host–pathogen interactions, and essential metal ions are often sequestered in tight-binding interactions, which allow for little to no metal exchange.^47^ Therefore, we hypothesize that Mg^2+^ is likely to be the metal used in vivo by *Pa*PepA to carry out catalytic function based on cellular abundance, especially on binding to the weaker site, considering a smaller difference in *K*_ACT_ as opposed to *K*_D_ when compared to Mn^2+^. Using the *K*_D_ values and kinetic parameters *k*_cat_/*K*_ACT_ for Mn^2+^ and Mg^2+^, respectively, we carried out a simulation using Kintek Global Explorer, illustrating a small percentage of turnovers proceeding while using Mn^2+^ as activating metal ion (∼1%), as opposed to Mg^2+^. More details on the simulation are available in Supporting Information Note 2.

### Metal Binding Stoichiometry and Thermodynamics

ITC was performed to determine the thermodynamics of metal binding. Mg^2+^ and Mn^2+^ were both investigated as ligands since Mn^2+^ displayed the highest *k*_cat_/*K*_ACT_ (Figure 3A) and Mg^2+^ is an abundant metal in the cell, which can activate *Pa*PepA (Figure 3D). The ITC data for both metals fit well to a model of one binding site per monomer, but *K*_D_ was much lower for Mn^2+^ at 13.6 ± 0.5 nM, as compared to 2.1 ± 0.3 μM for Mg^2+^. The number of metal atoms binding the protein was below 1 for both at 0.72 for Mn^2+^ and 0.68 for Mg^2+^. The titration curve indicates that *Pa*PepA has approximately 150 times greater affinity for Mn^2+^ than Mg^2+^.

*K*_ACT_ was 100 μM for Mn^2+^, and it was 3 mM for Mg^2+^ (Figure 3A, Table 1). The difference between *K*_D_ and *K*_ACT_ for Mn^2+^ prompted us to use EPR spectroscopy in solution as an analogous technique to probe the stoichiometry and affinity of the *Pa*PepA metal binding interaction. Data were fitted with a 1:0.9 (enzyme:Mn^2+^) binding model (Figure 3E). The best fit that could be obtained from this model showed a *K*_D_ of ∼174 nM. This is of similar magnitude to the values for Mn^2+^ binding obtained using ITC (Figure 3A,B and Table S7). EPR spectroscopy is sensitive to Mn^2+^ concentrations in the μM range and above. Thus, the uncertainty in *K*_D_ values lies in the hundreds of nM to μM range and any values in this system are to be taken as upper limits of *K*_D_ rather than precise estimates in the nM range are at the lower limit of detection by EPR.

Overall, these data indicate that *Pa*PepA has a tight-binding metal site with an approximately 1:1 binding stoichiometry. The reduced stoichiometry could be due to the presence of unfolded, aggregated, or inactive *Pa*PepA. However, the *K*_ACT_ values from Michaelis–Menten fits at pH 8.0 are comparatively much higher than the tight *K*_D_ values observed from ITC and EPR spectroscopy. Observed *K*_ACT_ values were in the micromolar region (∼100 μM) for MnCl_2_, and in the millimolar region for MgCl_2_ (∼3 mM). It is therefore unlikely that the occupation of this single tight-binding site is sufficient for *Pa*PepA activity.

To probe the second binding site, EPR spectroscopy was performed in a frozen solution in the presence of various ratios of PepA:Mn^2+^ (Figure 3F).^62^ A control lacking added *Pa*PepA showed a large well-resolved narrow-line signal for free Mn^2+^ (gray line). This characteristic signal disappeared, and a much broader signal was observed when Mn^2+^ was present at sub-stoichiometric concentrations when compared to *Pa*PepA, implying that the metal ion was binding the protein. When [metal ions] exceeded *Pa*PepA at 4:3 and 2:1 Mn^2+^:*Pa*PepA ratios, broad but resolved lines are visible between 200 and 300 mT. These are tentatively attributed to pairs of Mn^2+^, which are spatially close to one another. This signal only occurred when the metal ion concentration exceeded [*Pa*PepA], pointing toward one high-affinity metal binding site being occupied first, followed by a second lower affinity site. Only when there is an excess of metal over protein do we see occupation of the second site and this corresponding broad signal. Simultaneously, the signal of free aqueous Mn^2+^ is visible as soon as the metal concentration exceeded [*Pa*PepA], indicating the lower affinity for the binding of the second metal ion.

### PepA Requires Both Its Metal Binding Sites to Be Fully Occupied to Fulfill Its Catalytic Function

Crystal structures depicting two metals in the active site and the difference in magnitude for *K*_ACT_ and *K*_D_ values led us to hypothesize that metal occupancy in one site was not sufficient for activation. Importantly, care must be taken to interpret metal occupancy in structural data, as it can be influenced by crystallization conditions and not necessarily reflect protein activation state, metal binding stoichiometry, and catalysis.^49^

**Figure 4:**
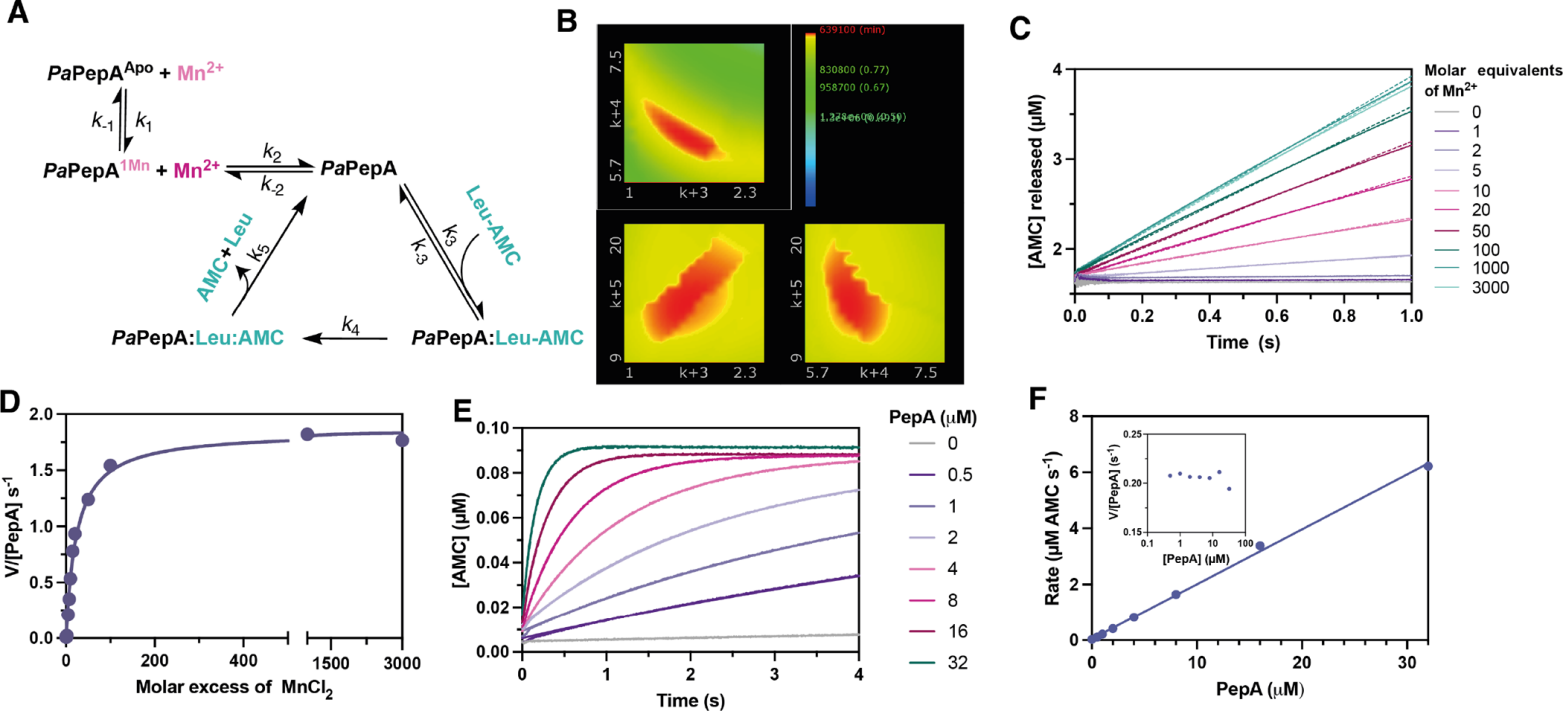
Pre-steady-state kinetics of *Pa*PepA breakdown of Leu-AMC. (A) Model used for data fitting. (B) Pairwise confidence contours for the *Pa*PepA reaction with MnCl_2_ and Leu-AMC. Plots were generated using the FitSpace editor in KinTek Global Explorer. The red color shows the area of best fit, and the *x* and *y* axes show the ranges for each rate constant. Plots show the dependence of the reciprocal of the normalized chi^2^ value versus each parameter pair. (C) Multiple turnover reactions of *Pa*PepA-mediated cleavage of Leu-AMC in the presence of different concentrations of [MnCl_2_]. Continuous lines = average trace across three replicates. Dotted line = linear regression fit. (D) *V*/[*Pa*PepA] vs [MnCl_2_] from the data in (A) fit to a hyperbolic curve. (E) Single turnover experiment for 0.1 μM Leu-AMC substrate vs increasing concentrations of *Pa*PepA. (F) Rate of AMC formation vs [*Pa*PepA], from data in (E). Fitted values are shown in Table 2. Details on data fitting are available on Supporting Information Note 4.

Pre steady-state multiple turnover (MTO) experiments were carried out in the presence of excess Leu-AMC while varying the ratio of MnCl_2_:*Pa*PepA (Figure 4). A broad range of metal:enzyme ratios was investigated to explore the difference between *K*_D_ and *K*_ACT_ for PepA-mediated catalysis when using Mn^2+^ (Figure 4C,D). No significant increase in signal was observed until the concentration of MnCl_2_ exceeded that of *Pa*PepA twofold, implying that both metal binding sites must be occupied for a cleavage reaction to occur. Beyond the 2:1 metal:protein ratio, reaction rates increased in a hyperbolic fashion with increasing [MnCl_2_] (Figure 4D). The half maximal rate was obtained in the presence of a 24-fold molar excess of metal, with a plateau reached at a 300-fold excess. The large excess of MnCl_2_ that is required to reach plateau suggests that one site binds Mn^2+^ weakly compared to the high-affinity site characterized by ITC and EPR spectroscopy and that metal readily dissociates from this weaker binding site. This means that a large molar excess of metal is required to reach saturation of the second site.

MTO traces collected in the presence of excess metal did not show a biphasic reaction. Lack of a visible burst phase suggests that a step after chemistry is not limiting *k*_cat_. MTO experiments using Leu-pNA instead of Leu-AMC as the substrate also did not show a visible burst, confirming that product release is not rate limiting for the *Pa*PepA-mediated cleavage of both substrates (Figure S12).

The absence of a burst of product formation points toward chemistry or a step preceding or coupled to chemistry being rate limiting to turnover. Single turnover kinetic studies of *Pa*PepA-catalyzed cleavage of Leu-AMC were performed to obtain information about the chemical step. In one syringe, *Pa*PepA was incubated with excess MnCl_2_. Leu-AMC was present in the other syringe. Figure 4F depicts the replot of the observed STO rates as a function of Leu-AMC substrate. To eliminate the contribution of bimolecular steps to the observed rate, substrate saturation must occur. However, across the range of enzyme concentrations tested, reaction rates continued to double even when with a 160-fold to 320-fold excess of protein over Leu-AMC (Figure 4E). We propose this could be due to fast release of Leu-AMC from *Pa*PepA, so its dissociation rate constant (*k*_–3_) is much faster than that of subsequent steps.

Global fitting of MTO and STO experiments yielded best-fitted values of 6.3 and 13.4 s^–1^ for *k*_4_ and *k*_5_, respectively (Figure 4A, Table 2). Together with the lack of a burst phase, data agree with a significant contribution of chemistry to the rate of steady-state turnover. Details on data fitting are available in Supporting Information Note 3.

### Viscosity effects

According to the presteady-state results, diffusional effects are not significantly limiting steady-state turnover. Subsequently, solvent viscosity effects were determined by measuring saturation curves at different concentrations of sucrose while varying Leu-pNA and Mn^2+^.^50,51^

*k*_cat_/*K*_M_ for Leu-pNA and *k*_cat_/*K*_ACT_ for Mn^2+^ show no significant deviation with different concentrations of sucrose, which is consistent with substrate and metal binding not being limited by their rate of diffusion into the active site^51^ (Table S8). A slope between 0 and 1 (observed 0.2 ± 0.1), as seen on *k*_cat_, suggests that the rate examined here may be slightly limited by product release. Using 5% PEG-8000 as a macroviscogen had no impact on the saturation curves (Figure S13), which verified that the previously observed effect was simply due to sucrose acting as a microviscogen. These data agree with MTO and STO experiments, since fitted values for peptide bond hydrolysis and product release were of similar magnitude, but with product release twice as fast.

### SKIEs

**Figure 5:**
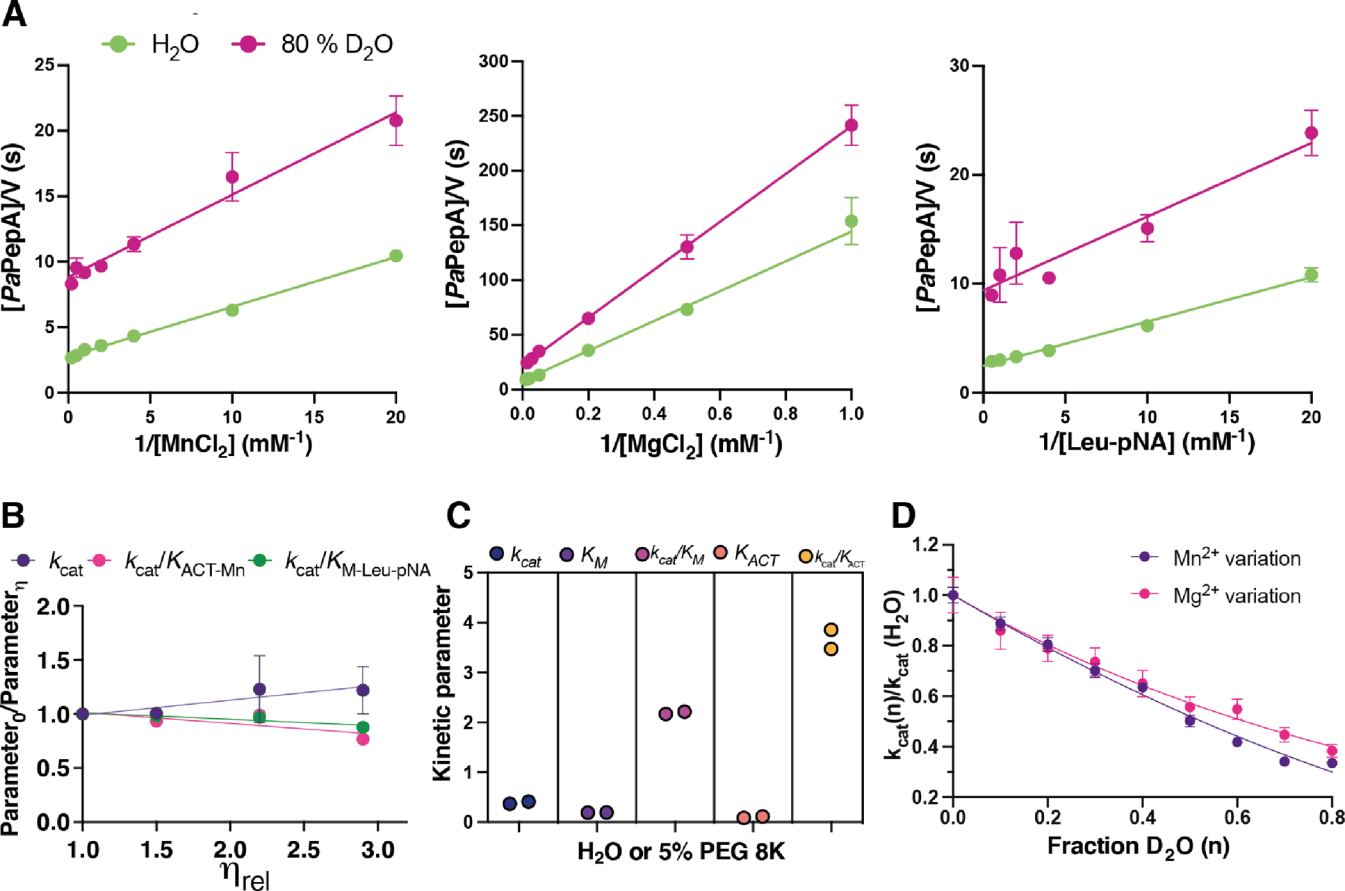
Solvent effects. (A) Solvent kinetic isotope effects for *Pa*PepA. Lineweaver–Burk plots for variation of MnCl_2_ (left), MgCl_2_ (center), and Leu-pNA (right). Data are mean for three replicates ± SEM. (B) Solvent viscosity effects on *k*_cat_ and *k*_cat_/*K*_M_ for Leu-pNA and Mn^2+^, conducted while varying the concentration of sucrose (C) or with 5% PEG-8000. Data are mean for three replicates ± SEM. (D) Proton inventory for *Pa*PepA *k*_cat_ values while varying MnCl_2_ or MgCl_2_ at saturating concentrations of Leu-pNA. Data best fit to a model accounting for two transition state protons with equal fractionation factor (eq 3). Data are mean for three replicates ± SEM.

As highlighted in Scheme 3, proton transfer steps are crucial for *Pa*PepA-catalyzed peptide bond hydrolysis. To probe the rate-limiting nature of proton transfer steps, SKIEs on *Pa*PepA were determined by monitoring cleavage of Leu-pNA while varying the concentration of either MnCl_2_, MgCl_2_, or Leu-pNA (Figure 5A). Reactions were performed in aqueous buffer in water or in 80% v/v D_2_O. The viscosity study performed above with sucrose revealed no effect on *k*_cat_/*K*_M_ for Leu-pNA, which is important as the relative viscosity of D_2_O is larger than H_2_O. SKIEs on *k*_cat_/*K*_ACT_ were not interpreted mechanistically for experiments involving [metal] variation due to the lack of a well-defined plateau in the pH curve for this parameter (Figure S8B). Small changes in pH that occur because of an increase in [D_2_O] could therefore have large impacts on *k*_cat_/*K*_ACT_. For curves varying Leu-pNA, pH 8.0 was used to minimize the effect of small pH variations on the *k*_cat_/*K*_M_ parameter as there is a well-defined plateau (Figure S8B). SKIEs (^D2O^*k*_cat_ and ^D2O^*k*_cat_/*K*_M_, ^D2O^*k*_cat_/*K*_ACT_) are reported on Table 3. When varying Mn^2+^, ^D2O^*k*_cat_/*K*_ACT-Mn_ was 1.9 ± 0.2 and ^D2O^*k*_cat_ was 3.9 ± 0.1, while when varying Mg^2+^ at fixed concentrations of Leu-pNA, ^D2O^*k*_cat_/*K*_ACT-Mg_ was 2.3 ± 0.1 and ^D2O^*k*_cat_ was 3.1 ± 0.1. Varying Leu-pNA concentrations of Mn^2+D2O^*k*_cat_/*K*_M-LeupNA-Mn_ were 2.2 ± 0.5. All values for SKIEs are listed in Table 3. Normal SKIEs suggest that proton transfer is at least partially rate limiting under these conditions. SKIEs of similar magnitude for ^D2O^*k*_cat_/*K*_ACT-Mn_ and ^D2O^*k*_cat_/*K*_ACT-Mg_ as well as ^D2O^*k*_cat-Mn_ and ^D2O^*k*_cat-Mg_ indicate poorer metal activation with Mg^2+^ to be driven by poor affinity on the second metal binding site rather than by an increase in the energy barrier for steps involving solvent exchangeable groups in the reaction coordinate.

**Table 3:** Summary of Solvent Deuterium Kinetic Isotope Effects

**SKIE**	**value**	**measured**
^D2O^*k*_cat-Mn_	3.9 ± 0.1	varying Mn
^D2O^*k*_cat_/*K*_ACT-Mn_	1.9 ± 0.2	varying Mn
^D2O^*k*_cat-Mg_	3.1 ± 0.1	varying Mg
^D2O^*k*_cat_/*K*_ACT-Mg_	2.3 ± 0.1	varying Mg
^D2O^*k*_cat_/*K*_M-LeupNA-Mn_	2.2 ± 0.5	varying Leu-pNA, fixed Mn

Proton inventory studies were carried out to obtain more in-depth information about the number and nature of protons giving rise to the sizable observed ^D2O^*k*_cat_ (Figure 5D). Considering the best fit to experimental data and calculated SKIE in comparison to measured SKIE, a model with a single proton [reactant (RS) or transition state (TS)] is not compatible (Table S9). The simplest model that best fit the data was of two identical fractionation factors for two protons participating in the transition state. Previously, metal-coordinated water molecules were determined to have inverse fractionation factors (in the range of 0.7–1.0 for Mn^2+^ and Mg^2+^).^52,53^ These metal-bound water molecules are RS protons and therefore could give rise to inverse SKIEs. We propose the protons giving rise to the SKIE are not derived from the metal-bound water because (1) the ^D2O^*k*_cat_ is normal and sizable, and the contribution of an inverse fractionation factor is an equilibrium isotope effect,^54^ which in the *Pa*PepA-catalyzed reaction would have taken place and be established when ^D2O^*k*_cat_ is determined, and (2) proton inventory data fitting including reactant protons did not result in reliable fits. The precise nature of the two transition state protons identified is speculative but could involve the protonation of the peptide substrate N-terminus and/or protonation steps taking place during tetrahedral intermediate dissolution, as depicted on Scheme 3.

In comparison to other aminopeptidases, the ^D2O^*k*_cat_ observed for *Pa*PepA are larger and present a curved proton inventory indicative of more than one proton contributing to the observed SKIE. In the reaction catalyzed by the methionine aminopeptidase from *E. coli*,^55^ a ^D2O^*k*_cat_ of 1.6 was determined and attributed to a single proton, while the aminopeptidase from *Aeromonas proteolytica* displayed a curved proton inventory indicative of two protons and a ^D2O^*k*_cat_ of 2.8.^40^

### Structure of *Pa*PepA

#### General Features

PepA crystal structures were determined to a resolution of 1.8 Å (apo enzyme, no added metal, PDB 8PZO), 1.97 Å (Mn^2+^ bound, PDB 8PZY), and 1.7 Å (with Bestatin inhibitor and Mn^2+^ bound, PDB 8PZM). Major features of the protein structure are identical in all three crystal forms. RMSD between apo and bestatin-bound *Pa*PepA is 0.21 Å, and the RMSD between the apo structure and Mn^2+^ bound *Pa*PepA is 0.21 Å. The apo enzyme occupied the metal binding sites, with likely Na^+^ ions present in the purification buffer. Solved structures reveal the presence of a *Pa*PepA hexamer, which displays high similarity with *E. coli*, bovine lens, and *P. putida* PepA hexamers (RMSD between 0.4 and 0.5 in comparison to *Pa*PepA). *Pa*PepA also exists as a hexamer in solution, which was independently determined by dynamic light-scattering analysis (Figure S14).

#### Metal-Bound PaPepA

Mn^2+^ was pentacoordinated in unliganded enzyme and hexacoordinated when bound to bestatin, but distances from coordinating residues remained the same, showing little to no distortion in the metal binding site and coordinating residues brought upon by ligand binding. Despite differences in metal preference between enzymes, active site residues and metal coordination on the first and second shells^56^ are identical to *Pp*PepA. CheckmyMetal^57^ revealed that the metal binding sites for *Pa*PepA are optimal for Mn^2+^. If the metal is replaced by Zn^2+^, a large RMSD of observed geometry angles (ligand–metal–ligand angles) compared to ideal geometry is seen, further arguing against Zn^2+^ as a viable metal.

#### Bestatin-Bound PaPepA

**Figure 6:**
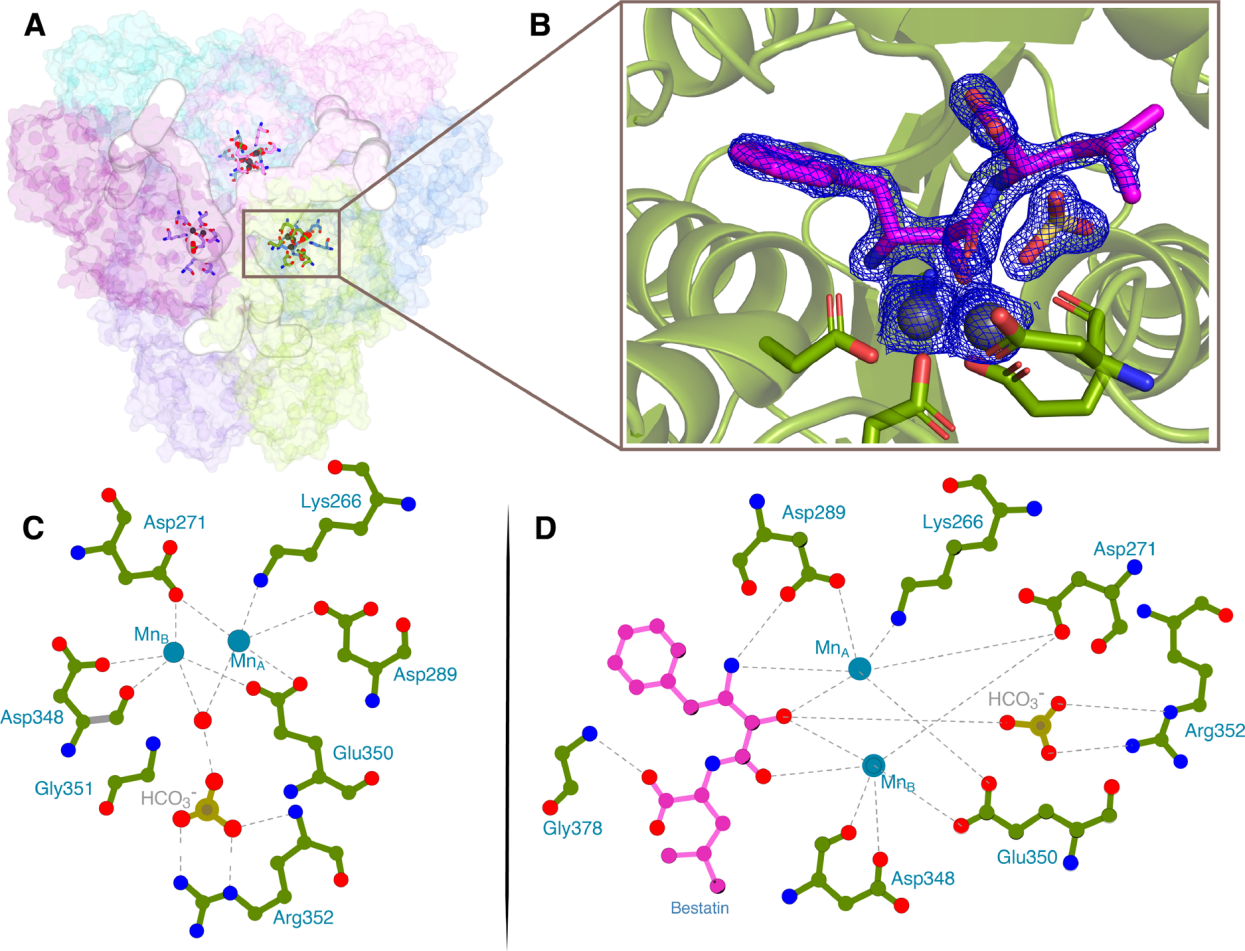
Structure of *Pa*PepA. (A) Hexameric *Pa*PepA structure bound to bestatin. (B) 2Fo-Fc map at 3σ depicting bestatin and two Mn^2+^ binding sites. (C) Interaction network of protein with metal ligands (Mn_A_ and Mn_B_), depicting a water molecule positioned between metal centers. Figure S15 shows the 2Fo-Fc map for this model. (D) Interactions between bestatin (pink) and metal ligands (Mn_A_ and Mn_B_). Dotted lines depict atoms within a 3 Å distance. Individual distances are available in the Supporting Information.

Bestatin is a modified peptide possessing the sequence H-βAla(2S–OH,3R-Bn)-Leu-OH and a tight inhibitor of proteases and aminopeptidases.^58^ In the structure with *Pa*PepA, bestatin is part of an extensive network of hydrogen bonding interactions as well as direct interaction with one of the metal atoms (Figure 6B,D). The amino terminus of bestatin interacts with a Mn^2+^ atom as well as with E289, and the hydroxyl group is in hydrogen bonding distance to K266.

Analysis of tunnels and cavities in *Pa*PepA using Caver^34^ revealed six possible tunnels leading to a central catalytic chamber, with three separate paths linking the protein active sites and metal binding centers to the protein surface. In our structures, tunnels contain a narrow portion closer to the active site (3.5 Å diameter on average at their narrowest part), therefore, excluding the possibility of an extensively folded peptide or protein acting as a substrate. Additionally, the location of tunnels highlights the presence of one viable path for peptide binding per *Pa*PepA in the interface between two monomers.

### Significance for *Pa*PepA Regulation and Metalloaminopeptidase Catalysis

Our characterization of the intracellular aminometallopeptidase from *P. aeruginosa* PaPepA combined pH and viscosity studies, SKIEs, kinetics in the steady state and presteady state with different substrates, EPR spectroscopy, ITC, and X-ray crystallography, revealing a thorough and complex mechanism by which this aminopeptidase is regulated and acts on diverse substrates. We put forward a mechanism for *Pa*PepA-catalyzed reactions in which bicarbonate does not play a role in acid–base catalysis. Both peptide bond hydrolysis and product release contribute to the rate of steady-state turnover. Our work revealed that the activity of *Pa*PepA is regulated in terms of metal activation, substrate selection, and availability as well as abundance of leucine, phenylalanine, and potentially other amino acids.

Tight regulation of the aminopeptidase function may be an important property for cell homeostasis. Aminopeptidases participate in nutrient recycling and alter the N-terminal sequences of proteins and peptides, implicating these enzymes in the regulation of protein stability and half-life.^59^ Previously, mutations aimed at altering the *Pa*PepA hexameric structure led to a slow growth phenotype in *P. aeruginosa*.^60^ The *Pa*PepA quaternary structure reveals that peptides can only enter the narrow tunnels leading to the metal center and catalytic residues if fully unfolded; therefore, peptide sequence and fold play a role in substrate acceptance. Importantly, metal activation is only achieved at high concentrations of Mn^2+^ or Mg^2+^, with great molar excess over *Pa*PepA. Because the intracellular concentration of Mn^2+^ is in the micromolar range, while concentration of Mg^2+^ is in the millimolar range, it is possible that, despite lower activation levels achieved, Mg^2+^ acts as the “physiological metal”, leading to *Pa*PepA activity and peptide hydrolysis. The presence of a high-affinity metal binding site, for which occupancy is close to 100%, and a low-affinity metal binding site, for which occupancy is low and dependent on metal identity and availability, provides an additional nuanced form of catalytic regulation. This highlights the importance of evaluating *K*_D_ and *K*_ACT_ parameters for metalloenzymes, as they reveal crucial mechanistic features of metal activation and catalysis in metalloenzymes.

We dissected how the metalloaminopeptidase *Pa*PepA catalyzes peptide bond hydrolysis, impacting future work characterizing,^16^ designing,^17^ and inhibiting^18^ metalloenzymes, which correspond to half of all enzymes.^61^

## Data Availability

The EPR data underpinning this publication will be accessible at DOI: 10.17630/94fc28c1-c364-4dde-b5b7-8deb5d68ff4c.^62^

## Supporting Information Available

Protein sequences for PaPepA; simulations using Kintek Global Explorer for processive × distributive models; simulations using Kintek Global Explorer for Mn × Mg utilization; derivation of kinetic constants *k*_cat_, *k*_cat_/*K*_M-Leu-pNA_, and *k*_cat/KACT_; Kintek Global Explorer fitting of stopped flow data; PaPepA-mediated cleavage of Leu-pNA; peptide substrate screen; sequence logos for peptide sequences; degradation of AVLQSGFRKK-NH_2_; progress curves for degradation of parent peptide (AVLQSGFRKK-NH_2_); time-course assay investigating peptide hydrolysis; IC50 curves for amino acid products; stability and pH-rate profiles for PaPepA; catalytic mechanism proposals for PaPepA; PaPepA metal binding; isothermal titration calorimetry (ITC) of PaPepA with Mn^2+^ and Mg^2+^; multiple turnover stopped flow experiment with excess Leu-pNA; solvent macroviscosity studies; dynamic light scattering of PaPepA; Mn^2+^ binding site; primers for PaPepA cloning; pNA calibration curves at different pHs; ADH standard peptides following digestion with trypsin; SIR mass list and cone voltages; LC-MS -QDa mass detector settings; exponential fitted data for time courses with AVLQSGFRKK-NH_2_; average binding constants, concentrations, and stoichiometries from ITC; slopes when varying viscogen (sucrose) concentrations; SKIEs calculated by fitting proton inventory data; calculation of intrinsic solvent kinetic isotope effects; and crystallographic data (PDF)
Crystallographic data (CIF)
Crystallographic data (CIF)
Crystallographic data (CIF)
Crystallographic data (CIF)
Crystallographic data (CIF)
Crystallographic data (CIF)

## Supplementary Material

bi3c00420_si_001.pdf

bi3c00420_si_002.cif

bi3c00420_si_003.cif

bi3c00420_si_004.cif

bi3c00420_si_005.cif

bi3c00420_si_006.cif

bi3c00420_si_007.cif

## Accession Codes

*Pa*PepA – UniProt Q02RY8, pdb accession 8PZM, 8PZO, 8PZY

## Abbreviations

EPR: electron paramagnetic resonance
ITC: isothermal titration calorimetry
STO: single turnover
MTO: multiple turnover
LC-MS: liquid chromatography mass spectrometry
*Pa*PepA: leucine aminopeptidase from *Pseudomonas aeruginosa*
*Pp*PepA: leucine aminopeptidase from *Pseudomonas putida*
*Ec*PepA: leucine aminopeptidase from *Escherichia coli*
aa-pNA: amino acid-paranitroanilide;
aa-AMC: amino acid- 7-amido-4-methylcoumarin
