# Supplementary material for: Unveiling the Catalytic Mechanism of a Processive Metalloaminopeptidase
Source: Biochemistry. 2023 Nov 4;62(22):3188–205. doi: 10.1021/acs.biochem.3c00420 (PMC10666288; doi:10.1021/acs.biochem.3c00420)
Supplement: Supplementary file 1 — bi3c00420_si_001.pdf [file bi3c00420_si_001.pdf]

Supporting information

Unveiling the catalytic mechanism of a processive metalloaminopeptidase

Martha Clementine Simpson[1], Christopher John Harding[1], Ricardo Melo Czekster[2], Laura Remmel[3], Bela E. Bode[3], Clarissa Melo Czekster[1]*

1 University of St Andrews, School of biology, North Haugh, Biomolecular Sciences building, KY16 9ST, St Andrews, UK

2 Aston University, School of Computer Science and Digital Technologies, Department of Software Engineering and Cybersecurity, B4 7ET, Birmingham, UK

3 University of St Andrews, School of chemistry, North Haugh, Purdie building, KY16 9ST, St Andrews, UK

***To whom correspondence should be addressed:**

Clarissa Melo Czekster - School of Biology, University of St Andrews, Biomedical Sciences Research Complex, St Andrews, Fife KY16 9ST, U.K

Email: cmc27@st-andrews.ac.uk

Table of contents

Protein sequences for PaPepA ________________________ *S3*

Supporting notes: ________________________ *S4*

Supporting information note 1: Simulations using Kintek Global Explorer for processive x distributive models ________________________ S4

Supporting information note 2: Simulations using Kintek Global Explorer for Mn x Mg utilization __ S5

Supporting information note 3: Derivation of kinetic constants k_{cat}, $k_{cat}/K_{M\text{-Leu-pNA}}$ and k_{cat}/K_{ACT}. ____ S6

Supporting information note 4: Kintek Global Explorer fitting of stopped flow data ____________ S8

Supporting Figures ________________________ *S10*

Figure S1: PaPepA mediated cleavage of Leu- pNA. ________________________ S10

Figure S2: Peptide substrate screen. ________________________ S11

Figure S3: Sequence logos for peptide sequences. ________________________ S12

Figure S4: Degradation of AVLQSGFRKK-NH_2 ________________________ S12

Figure S5: Progress curves for degradation of parent peptide (AVLQSGFRKK-NH2) ____________ S13

Figure S6: Time course assay investigating peptide hydrolysis ________________________ S14

Figure S7: A) IC50 curves for amino acid products ________________________ S15

Figure S8: Stability and pH-rate profiles for PaPepA. (A) Stability studies for *Pa*PepA ______ S16

Figure S9: Catalytic mechanism proposals for PaPepA ________________________ S18

Figure S10: *PaPepA metal binding.* ________________________ S18

Figure S11: Isothermal titration calorimetry (ITC) of PaPepA with Mn2+ and Mg2+. ____________ S19

Figure S12: A) Multiple turnover stopped flow experiment with excess Leu-pNA. ____________ S20

Figure S13: Solvent macroviscosity studies ________________________ S21

Figure S14: Dynamic Light Scattering of PaPepA ________________________ S22

Figure S15: Mn2+ binding site. ________________________ S23

Supplementary Tables ________________________ *S24*

Table S1: Primers for PaPepA cloning ________________________ S24

Table S2: pNA calibration curves at different pHs. ________________________ S24

Table S3: ADH standard peptides following digestion with Trypsin ________________________ S25

Table S4: SIR Mass list and cone voltages ________________________ S27

Table S5: LC-MS - QDa mass detector settings: ________________________ S28

Table S6: Exponential fitted data for time courses with AVLQSGFRKK-NH_2 ____________ S29

Table S7: Average binding constants, concentrations, and stoichiometries from ITC ____________ S30

Table S8: Slopes when varying viscogen (sucrose) concentrations ________________________ S30

Table S9: SKIEs calculated by fitting proton inventory data ________________________ S31

Calculation of intrinsic Solvent kinetic isotope effects ________________________ S32

Table S10: Crystallographic data ________________________ S33

Protein sequences for PaPepA

Uniprot Q02RY8:

*Pa*PepA sequence prior to TEV cleavage:

```
MHHHHHHDYDIPTTENLYFQGGMEFLVKSVRPETLKTATLVLAVGEGRKLGASAKAVDDATGGAISAVLKRGDLA
GKVGQTLLLQSLPNLKAERVLLVGAGKERELGDRQYRKLASAVLSTLKGLAGADAALALGDLAVKGRGAHAKARL
LVETLADGLYVFDRYKSQKAEPLKLKKLTLLADKADSAAVEQGSKEAQAIANGMALTRDLGNLPPNVCHPTFLGE
QAKGLAKEFKSLKVEVLDEKKLRELGMGSFLAVAQGSDQPPRLIILQYNGAKKDQAPHVLVGKGITFDTGGISLK
PGLGMDEMKFDMCGAASVFGTFRAVLELQLPINLVGLLACAENMPSGGATRPGDIVTTMSGQTVEILNTDAEGRL
VLCDALTYAERFKPQSVIDIATLTGACIVALGSNTSGLMGNNEALVRQLLKAGEFADDRAWQLPLFDEYQEQLDS
PFADIANIGGPKAGTITAGCFLSRFAKKYHWAHLDIAGTAWISGGKDKGATGRPVPLLTQYLLERAK
```

*Pa*PepA sequence after TEV cleavage:

```
GGMEFLVKSVRPETLKTATLVLAVGEGRKLGASAKAVDDATGGAISAVLKRGDLAGKVGQTLLLQSLPNLKAERV
LLVGAGKERELGDRQYRKLASAVLSTLKGLAGADAALALGDLAVKGRGAHAKARLLVETLADGLYVFDRYKSQKA
EPLKLKKLTLLADKADSAAVEQGSKEAQAIANGMALTRDLGNLPPNVCHPTFLGEQAKGLAKEFKSLKVEVLDEK
KLRELGMGSFLAVAQGSDQPPRLIILQYNGAKKDQAPHVLVGKGITFDTGGISLKPGLGMDEMKFDMCGAASVFG
TFRAVLELQLPINLVGLLACAENMPSGGATRPGDIVTTMSGQTVEILNTDAEGRLVLCDALTYAERFKPQSVIDI
ATLTGACIVALGSNTSGLMGNNEALVRQLLKAGEFADDRAWQLPLFDEYQEQLDSPFADIANIGGPKAGTITAGC
FLSRFAKKYHWAHLDIAGTAWISGGKDKGATGRPVPLLTQYLLERAK
```

Supporting notes:

Supporting information note 1: Simulations using Kintek Global Explorer for processive x distributive models

Model used for processive reaction was as follows:
Pepa + AVLQSGFRKK = PepaAVLQSGFRKK = PepaVLQSGFRKK = PepaLQSGFRKK = PepaQSGFRKK = PepaSGFRKK = PepaGFRKK = PepaFRKK

Observables were:
PepaAVLQSGFRKK
PepaVLQSGFRKK
PepaLQSGFRKK
PepaQSGFRKK
PepaSGFRKK
PepaGFRKK
PepaFRKK
PepaRKK

Model used for processive reaction was as follows:
Pepa + AVLQSGFRKK = PepaAVLQSGFRKK
PepaAVLQSGFRKK = PepaVLQSGFRKK
PepaVLQSGFRKK = Pepa + VLQSGFRKK
Pepa + VLQSGFRKK = PepaVLQSGFRKK = PepaLQSGFRKK
PepaLQSGFRKK = Pepa + LQSGFRKK
Pepa + LQSGFRKK = PepaLQSGFRKK = PepaQSGFRKK
PepaQSGFRKK = Pepa + QSGFRKK
Pepa + QSGFRKK = PepaQSGFRKK = PepaSGFRKK
PepaSGFRKK = Pepa + SGFRKK
Pepa + SGFRKK = PepaSGFRKK = PepaGFRKK
PepaGFRKK = Pepa + GFRKK
Pepa + GFRKK = PepaGFRKK = PepaFRKK
PepaFRKK = Pepa + FRKK
Pepa + FRKK = PepaFRKK = PepaRKK

Observables were:
PepaAVLQSGFRKK + AVLQSGFRKK
PepaVLQSGFRKK + VLQSGFRKK
PepaLQSGFRKK + LQSGFRKK
PepaQSGFRKK + QSGFRKK
PepaSGFRKK + SGFRKK
PepaGFRKK + GFRKK
PepaFRKK + FRKK
PepaRKK + RKK

For both models, all binding steps were set with association rate constants = 1000$\mu M^{-1}s^{-1}$ and dissociation rate constants = 10s^{-1}.

Supporting information note 2: Simulations using Kintek Global Explorer for Mn x Mg utilization

The following model was used, where E is free enzyme, no metals bound, EMn1 or EMg1 are enzyme with one metal ion bound, EMn2 or EMg2 are enzyme with two metal ions bound, LeuMn is the product formed when Mn was the activating metal, LeuMg is the product formed when Mg was the activating metal.

E + Mn = EMn1
EMn1 + Mn = EMn2
EMn2 + LeupNA = EMn2LeupNA
EMn2LeupNA = EMn2 + LeuMn
EMn2 = Mn + EMn1
E + Mg = EMg1
EMg1 + Mg = EMg2
EMg2 + LeupNA = EMg2LeupNA
EMg2LeupNA = EMg2 + LeuMg
EMg2 = Mg + EMg1

For this simulation, the following conditions were used:
[Mn] = 0.32 mM
[Mg] = 150 mM
[Leu-pNA] = 100mM
[PaPepA] = 0.01mM
Constraints were used as indicated in the image below, based on values for K_D and k_{cat}/K_{ACT} (used to set a lower limit to association rate constants to metal ions). The product endpoints in the end of the simulation were 98.87% LeuMg, 1.12% LeuMn.

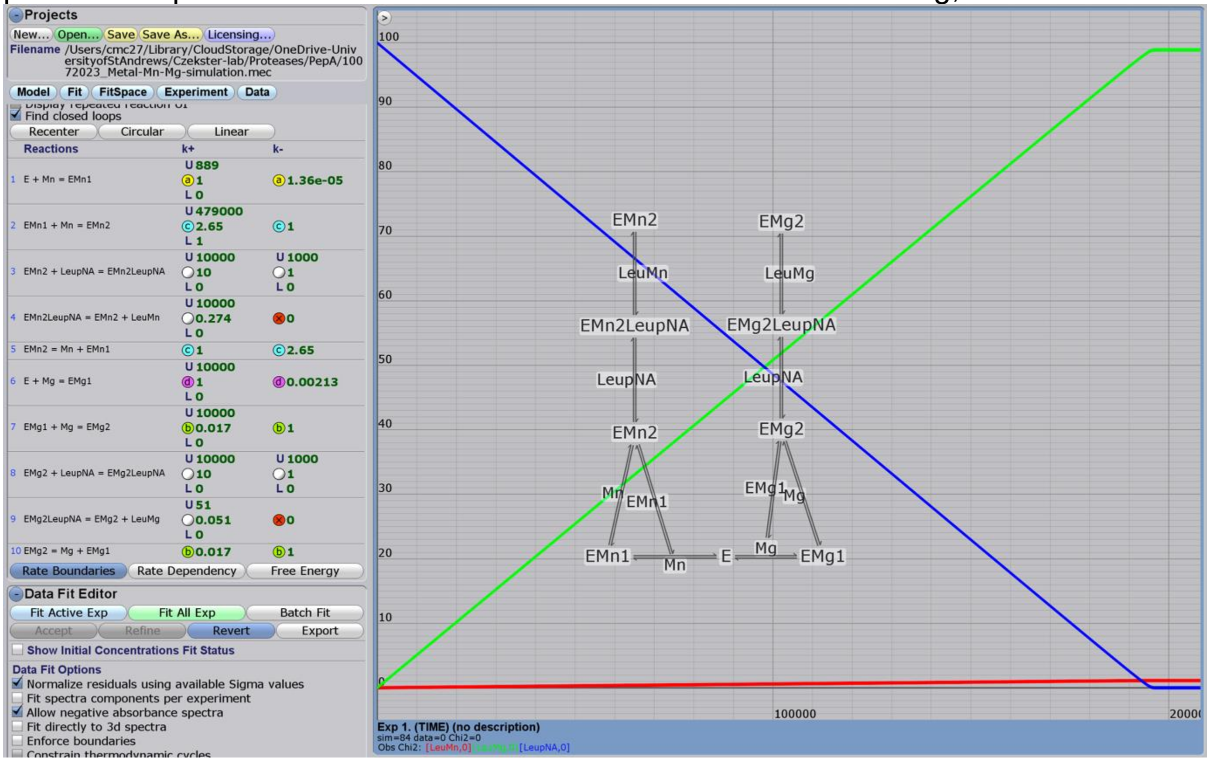

Supporting information note 3: Derivation of kinetic constants k_{cat}, $k_{cat}/K_{M\text{-Leu-pNA}}$ and k_{cat}/K_{ACT}.

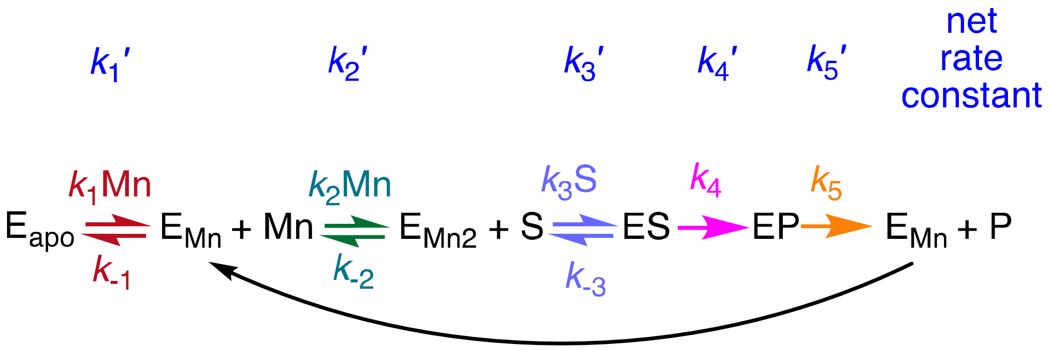

We used the net rate constants method[1] to derive equations defining k_{cat}, $k_{cat}/K_{M\text{-Leu-pNA}}$ and k_{cat}/K_{ACT}. This assumes that under steady state conditions chemistry and product release are irreversible. We also assumed PepA exists in a "1 bound metal" (E_{Mn}) during steady-state turnover due to the tight binding affinity to the first metal binding site. Binding to the weak site, however, needs to take place again between catalytic cycles, giving rise to the high value for K_{ACT}. In summary, the net rate constants for each enzyme species are:

$$k_5' = k_5$$

$$k_4' = k_4$$

$$k_3' = k_3 S\left(\frac{k_4}{k_4 + k_{-3}}\right) = \frac{k_3 k_4 S}{k_4 + k_{-3}}$$

$$k_2' = k_2 Mn\left(\frac{k_3'}{k_3' + k_{-2}}\right) = k_2 Mn\left(\frac{\frac{k_3 k_4 S}{k_4 + k_{-3}}}{\frac{k_3 k_4 S}{k_4 + k_{-3}} + k_{-2}}\right) = k_2 Mn\left(\frac{k_3 k_4 S}{k_3 k_4 S + k_{-2}(k_4 + k_{-3})}\right)$$

$$\frac{v}{[E_t]} = \frac{1}{\frac{1}{k_1'} + \frac{1}{k_2'} + \frac{1}{k_3'} + \frac{1}{k_4'} + \frac{1}{k_5'}} \rightarrow \frac{v}{[E_{Mn}]} = \frac{1}{\frac{1}{k_2'} + \frac{1}{k_3'} + \frac{1}{k_4'} + \frac{1}{k_5'}}$$

assuming free enzyme is non existing due to tight binding of metal to the first site, we eliminate k_1'. At saturating Mn and S

$$\frac{1}{k_2'} + \frac{1}{k_3'} \cong 0$$

$$k_{cat} = \frac{v}{[E_{Mn}]} = \frac{1}{\frac{1}{k_4'} + \frac{1}{k_5'}} = \frac{k_4 k_5}{k_4 + k_5} \qquad \text{(Equation S1)}$$

then

For k_{cat}/K_{ACT} with saturating Substrate:

$$\frac{\frac{v}{[E_{Mn}]}}{Mn} = \frac{k_{cat}}{K_{ACT}} = \frac{k_2'}{Mn} = \frac{k_2 k_3 k_4 S}{k_3 k_4 S + k_{-2}(k_4 + k_{-3})} \qquad \text{(Equation S2)}$$

For comparison, the affinity to the first metal site is defined by

$$K_D = \frac{k_{-1}}{k_1}$$

For k_{cat}/K_M with saturating Mn:

$$\frac{\frac{v}{[E_{Mn}]}}{S} = \frac{k_{cat}}{K_M} = \frac{k_3'}{S} = \frac{k_3 k_4 S}{k_4 + k_3} \qquad \text{(Equation S3)}$$

Supporting information note 4: Kintek Global Explorer fitting of stopped flow data

Both MTO and STO pre-steady-state data were fit according to the mode described in Figure 4A with scaling factors for each fluorescent species (EP and P) to account for differences in AMC fluorescence whilst in complex with *Pa*PepA versus free in solution. Scaling factors were different across STO and MTO experiments as both were carried out using different detector voltages.

The model that best fitted acquired data was as below, where F is metal free enzyme, E1 is PaPepA with one Mn^{2+} ion, E is PepA with two Mn^{2+} ions, S is Leu-AMC and P is Leucine:

F + Mn = E1

E1 + Mn = E

E + S = ES

ES = EP

EP = E + P

Observable: c*EP + d*P

For fitting, k_1 and k_{-1} ratios were constrained (linked) based K_D for Mn^{2+} binding as measured by ITC (12nM). Initially, k_2 and k_{-2} were allowed to vary without constraints, and values best fitted as well as Fitspace lower and upper boundaries are reported in Table 2. The value for k_1 was best fitted as diffusion limited, and therefore was fixed in further fitting cycles. The best fitted value for k_{-2} was high and unconstrained, and therefore also fixed at 1000 s^{-1} for further fitting cycles. k_3, k_{-3}, k_4, and k_5 were not constrained. Multiple iterations of fitting were performed, and best fit values recorded. k_1, k_2, k_3, k_{-3}, k_4, and k_5 were evaluated using FitSpace Explorer to generate confidence intervals for each of these rate constants. Figure 4B shows the 3-dimensional surfaces generated with Fitspace data analysis, displaying the extent to which any two parameters can co-vary and produce a fit that is well constrained by data. Fitting data to this 5-step mechanism and evaluating the fit showed a very low rate constant for k_3. Following metal binding, *Pa*PepA must bind its substrate- Leu AMC or Leu-pNA - which occurs quickly but with a fast k_{-3}, likely causing the lack of a plateau in observed rate constants for the STO experiment. The lack of a visible burst in the MTO experiments suggests that product release is not rate limiting, which is supported by the calculated values for k_4 and k_5 (Figure 4B).

Supporting Figures

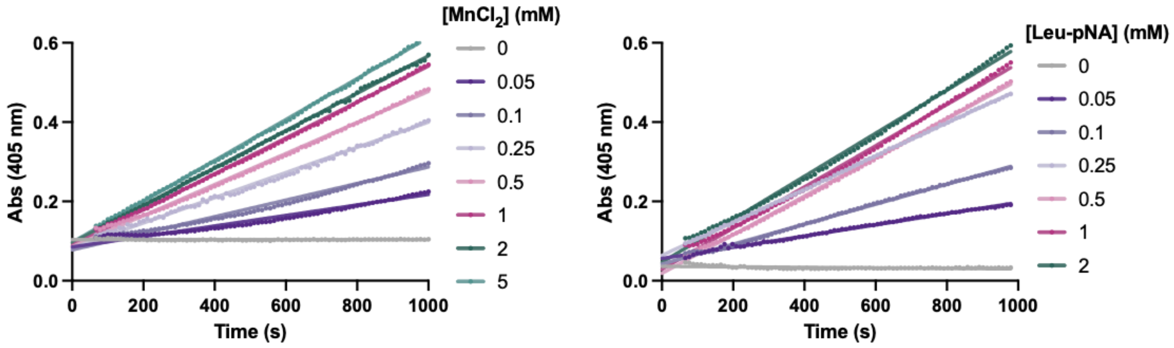

Figure S1: PaPepA mediated cleavage of Leu- pNA. Representative raw data for an aa-pNA substrate. Left: Raw data for cleavage of Leu-pNA when [Mn^{2+}] is varied and Leu-pNA is present in excess. Right: Raw data when [Leu-pNA] is varied and $MnCl_2$ is present in excess.

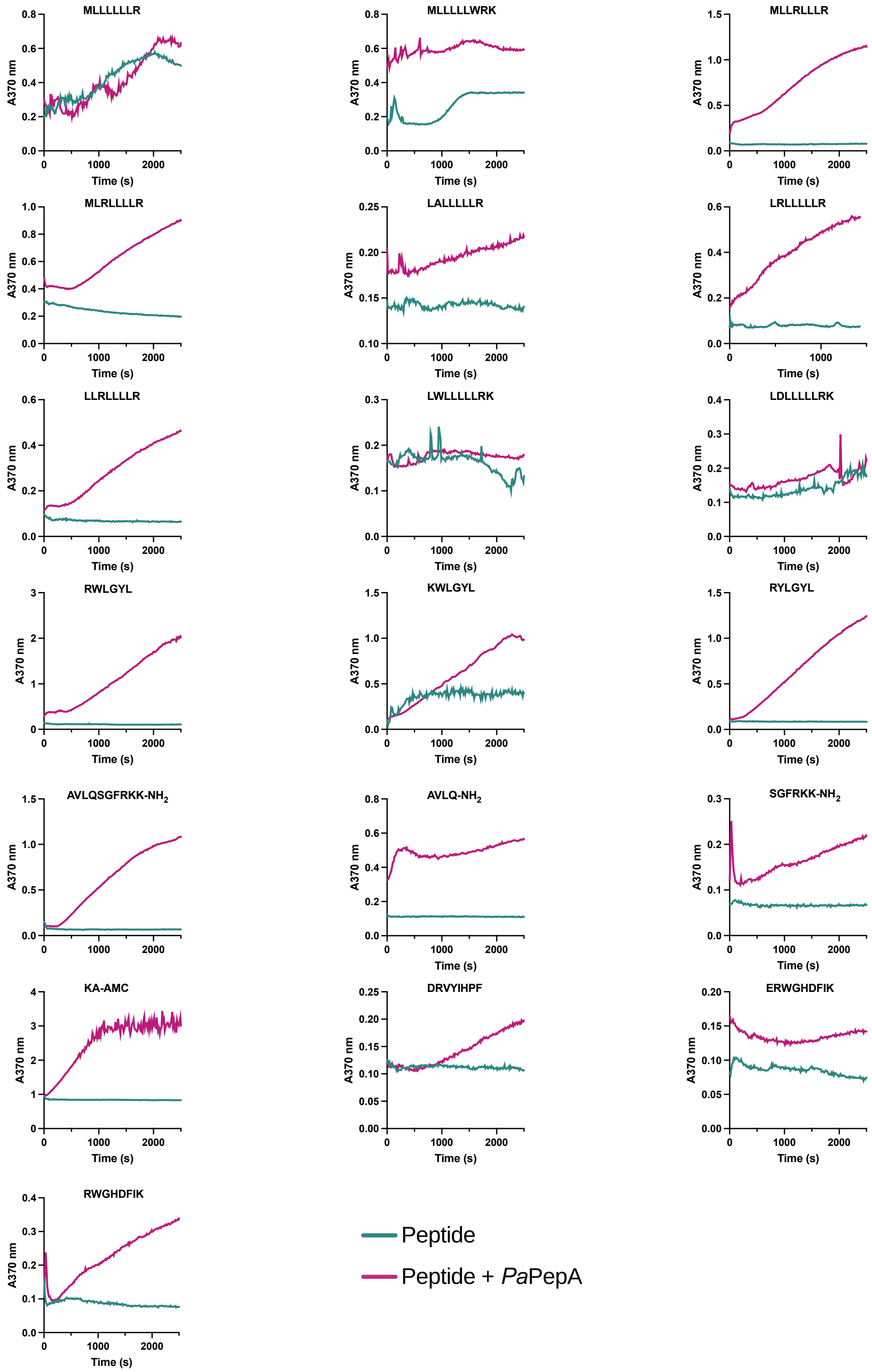

Figure S2: Peptide substrate screen. Reactions were monitored at 370 nm during incubation of *Pa*PepA with peptide, HRP, LAAO, and TMB.

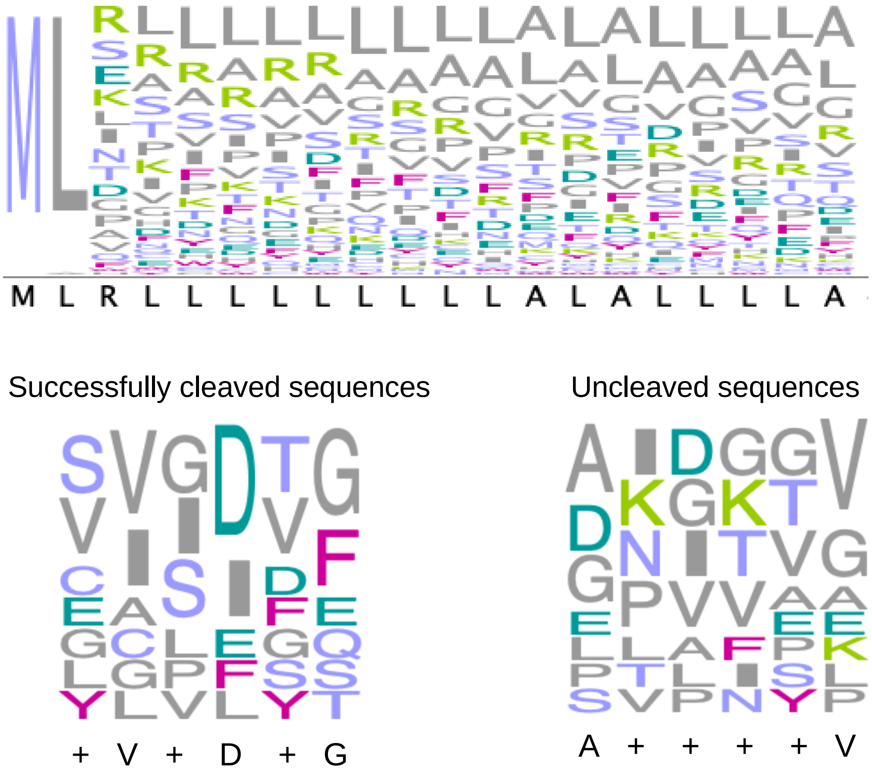

Figure S3: Sequence logos for peptide sequences. Top: Logo resulting from analysis of sequences from the P. aeruginosa PA14 proteome containing N-terminal methionine and leucine residues. Bottom: Logo from ADH peptide cleavage showing the first six residues of (left) *Pa*PepA-cleaved peptides, (right) uncleaved peptides.

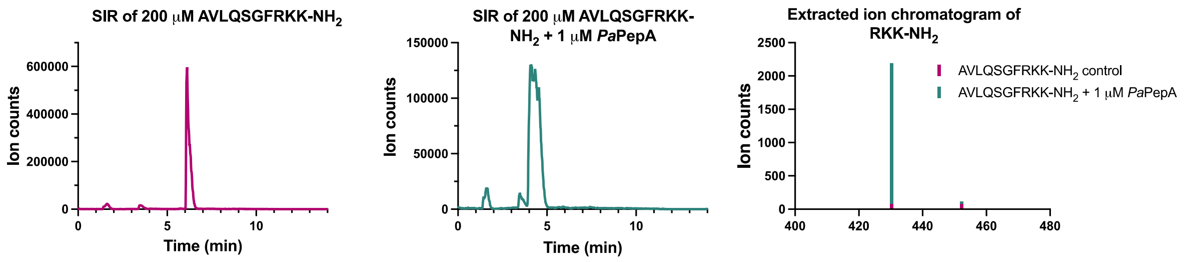

Figure S4: Degradation of AVLQSGFRKK-NH_2 to a tripeptide following incubation with 1 μM *Pa*PepA. (A) and (B) – SIRs monitoring AVLQSGFRKK-NH_2 and its degradation products when incubated in the absence (A) and presence (B) of *Pa*PepA for 6 hours, respectively. (C) Extracted ion chromatogram of RKK-NH_2 degradation product (+H^+ and +Na^+) showing increased abundance in the sample incubated with *Pa*PepA.

A

B

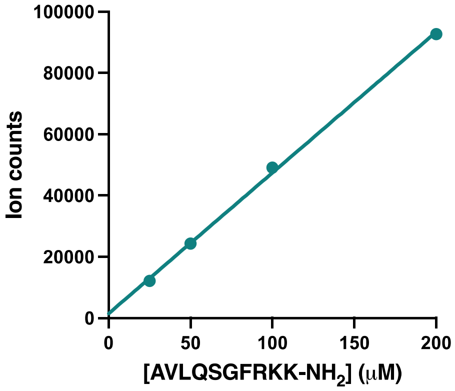

C

D

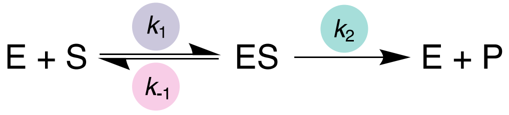

$k_{cat} = k_2$

$K_M = (k_2 + k_{-1}) / k_1$

$k_{cat}/K_M = (k_1 * k_2) / (k_2 + k_1)$

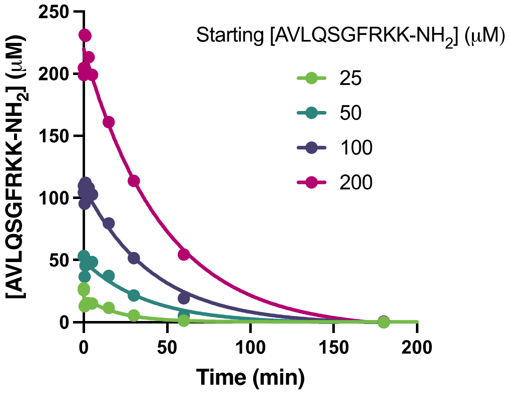

Parameter	Fitted number
k_1	0.040 ± 0.014
k_{-1}	0.0000095
k_2	4.59 ± 1.68
k_{cat}	4.59 min^{-1}
K_M	115 μM
k_{cat}/K_M	0.040 min μM^{-1}

Figure S5: **Progress curves for degradation of parent peptide (AVLQSGFRKK-NH2)** alongside Kintek fitted rate constants and calculated kinetic parameters. (A) Calibration curve of ion counts against concentration of parent peptide. (B) Decreasing concentrations of parent peptide throughout the experiment. Data were fit to a 1 phase exponential decay. (C) Scheme of the model used for fitting in Kintek Explorer, and how these parameters were used to calculate k_{cat}, K_M, and k_{cat}/K_M. Data in (D) Table shows the fitted and calculated kinetic parameters.

Prior to fitting, substrate peaks were integrated and slope of calibration curve used as a scaling factor for fitting. For data fitting using Kintek Global Explorer, the following model and assumptions were made:

Model: E + S = ES -> E+P
Observables:
Observable 1: AVLQSGFRKK-NH_2
Observable 2: a*E:VLQSGFRKK + VLQSGFRKK
a = 459.3 (as per calibration curve)

Assumptions:
- That cleavage (conversion of ES-> E+P) is irreversible, so $k_{-2} = 0$
- Because the observable monitored is substrate decay, presence of intermediate peptides as cleavage progresses were not included.

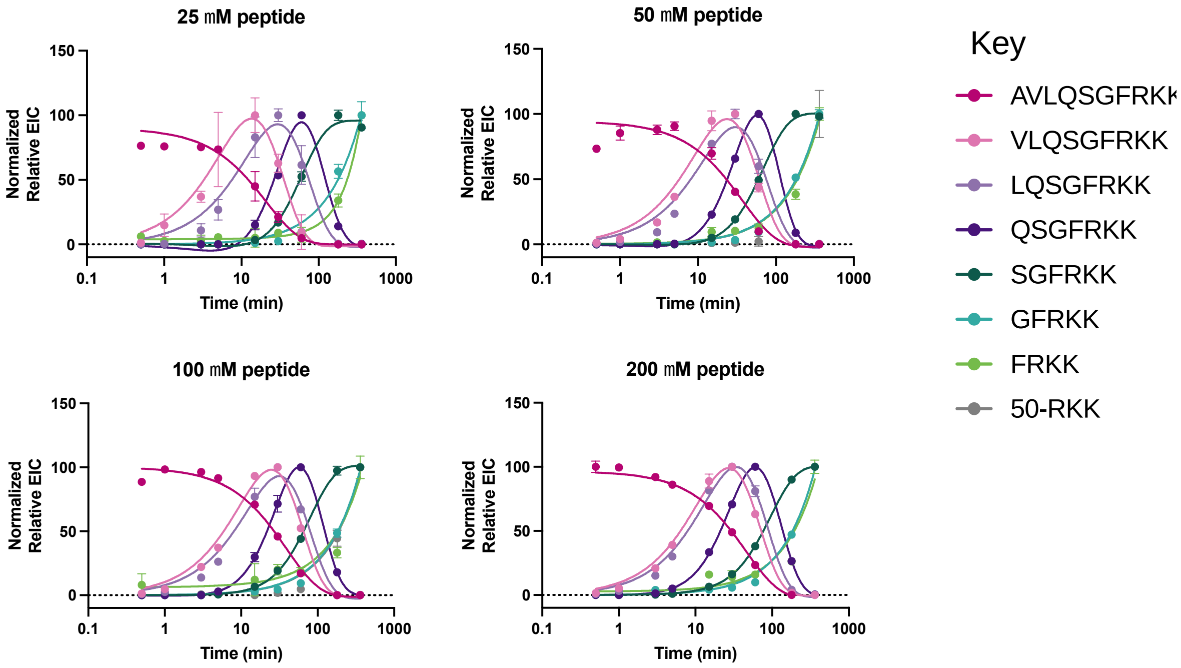

Figure S6: Time course assay investigating peptide hydrolysis using LC-MS to detect peptide fragments of reaction with 25, 50, 100, and 200 µM peptide and 1 µM *Pa*PepA quenched at different timepoints. Each datapoint was measured in duplicate and the data were fit to single (AVLQSGFRKK-NH_2), double (VLQSGFRKK, LQSGFRKK, SGFRKK, GFRKK, FRKK, and RKK), or triple (QSGFRKK-NH_2) exponential equations using GraphPad Prism.

Equations used for analytical fitting:

$$Y = (Y_0 - \text{Plateau})*e^{-k*x} + \text{Plateau}$$

$$Y = Y_0 + (\text{Plateau}_1 - Y_0)*(1 - e^{-k1*x}) + (\text{Plateau}_1 - \text{Plateau}_2)*(1 - e^{-k2*x}))$$

$$Y=Y_0 + (\text{Plateau}_1)*(1 - e^{-k1*x}) + (\text{Plateau}_2)*(1 - e^{-k2*x}) + (\text{Plateau}_3)*(1 - e^{-k3*x})$$

Where:
X: Time
Y: Y starts at Yo, then goes up to Plateau with one phase.
Y_0 and Amp: Same units as Y
k: Rate constant in units that are the reciprocal of the X axis units.

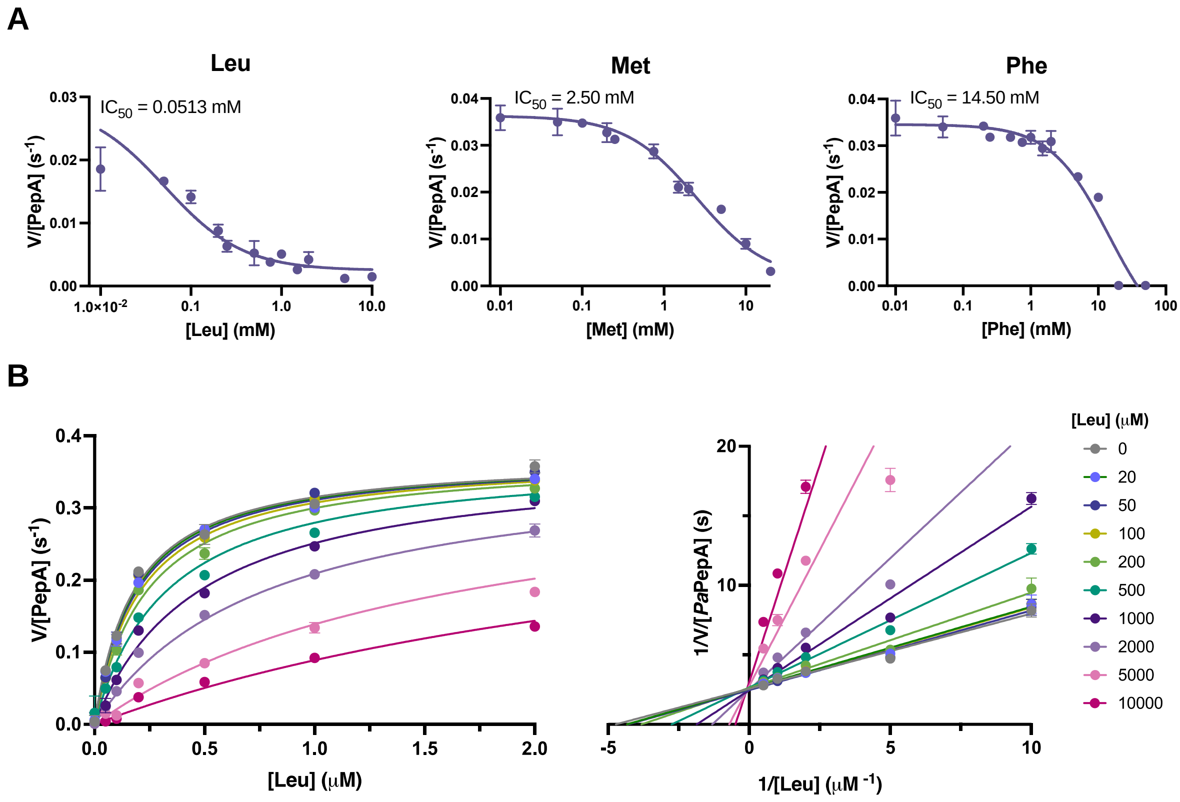

Figure S7: A) IC50 curves for amino acid products of *Pa*PepA mediated catalysis whilst in the presence of different concentrations of leucine, methionine, and phenylalanine. Leu-pNA (left), Met-pNA (center) and Phe-pNA (right) were all present at 237 μM = ~ K_M. $MnCl_2$ was present in excess. Error = SEM across three replicates. B) Michaelis Menten and Lineweaver-burke plots using different fixed concentrations of Leucine as an inhibitor. Data were best fit to a competitive inhibition model yielding K_i = 601.0 μM

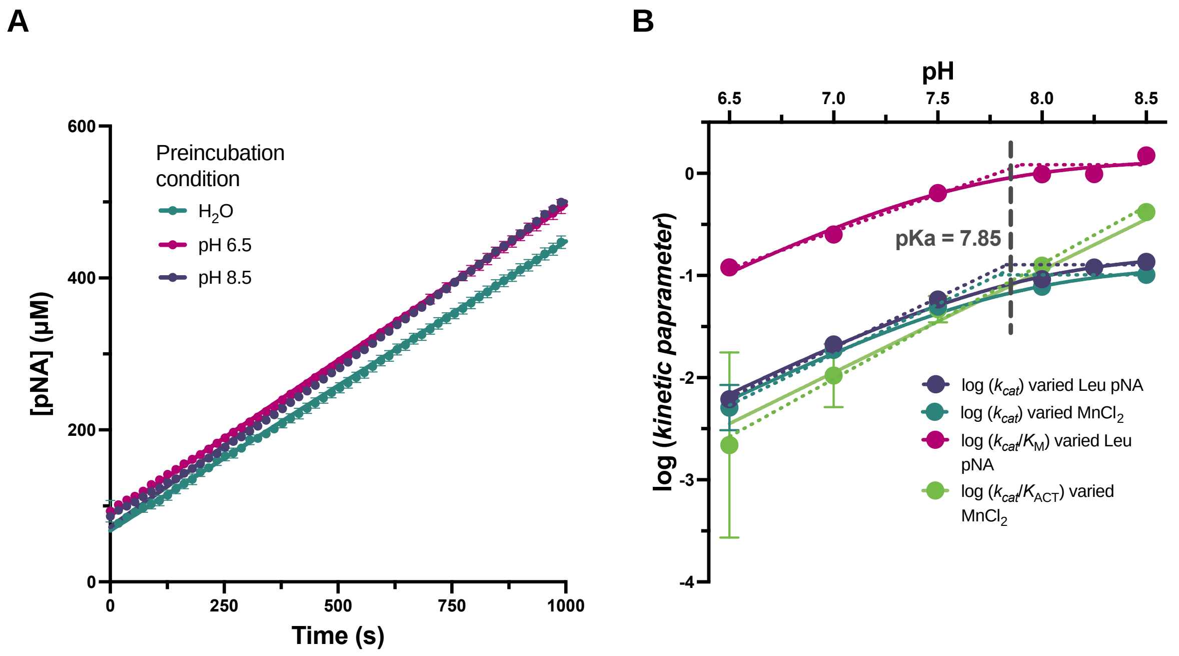

Figure S8: Stability and pH-rate profiles for PaPepA. (A) Stability studies for *Pa*PepA preincubated in mixed buffer at pH 6.5 (pink) or 8.5 (purple) for 1 hour prior to dilution into a standard reaction mixture (100 µM HEPES, 50 mM KCl, pH 8.0). Control (teal) represents *Pa*PepA incubation in H_2O for 1 hour. Reaction was carried out with Leu-pNA and $MnCl_2$ present at saturating conditions (2 mM and 3 mM respectively). Error bars represent SEM across three replicates. (B) pH dependence of log (k_{cat}) and log (k_{cat}/K_M) for varied Leu-pNA (purple and pink, respectively) and log(k_{cat}) and log(k_{cat}/K_{ACT}) for $MnCl_2$ (teal and green lines, respectively) examined using a mixed buffer system. Data were obtained at 25 °C in H_2O whilst varying the concentration of one reaction component with the other in excess. Data are fit to a 1-proton dependence equation[2], shown as mean values ± SEM across three replicates. Errors for k_{cat}/K_M and k_{cat}/K_{ACT} were propagated appropriately.

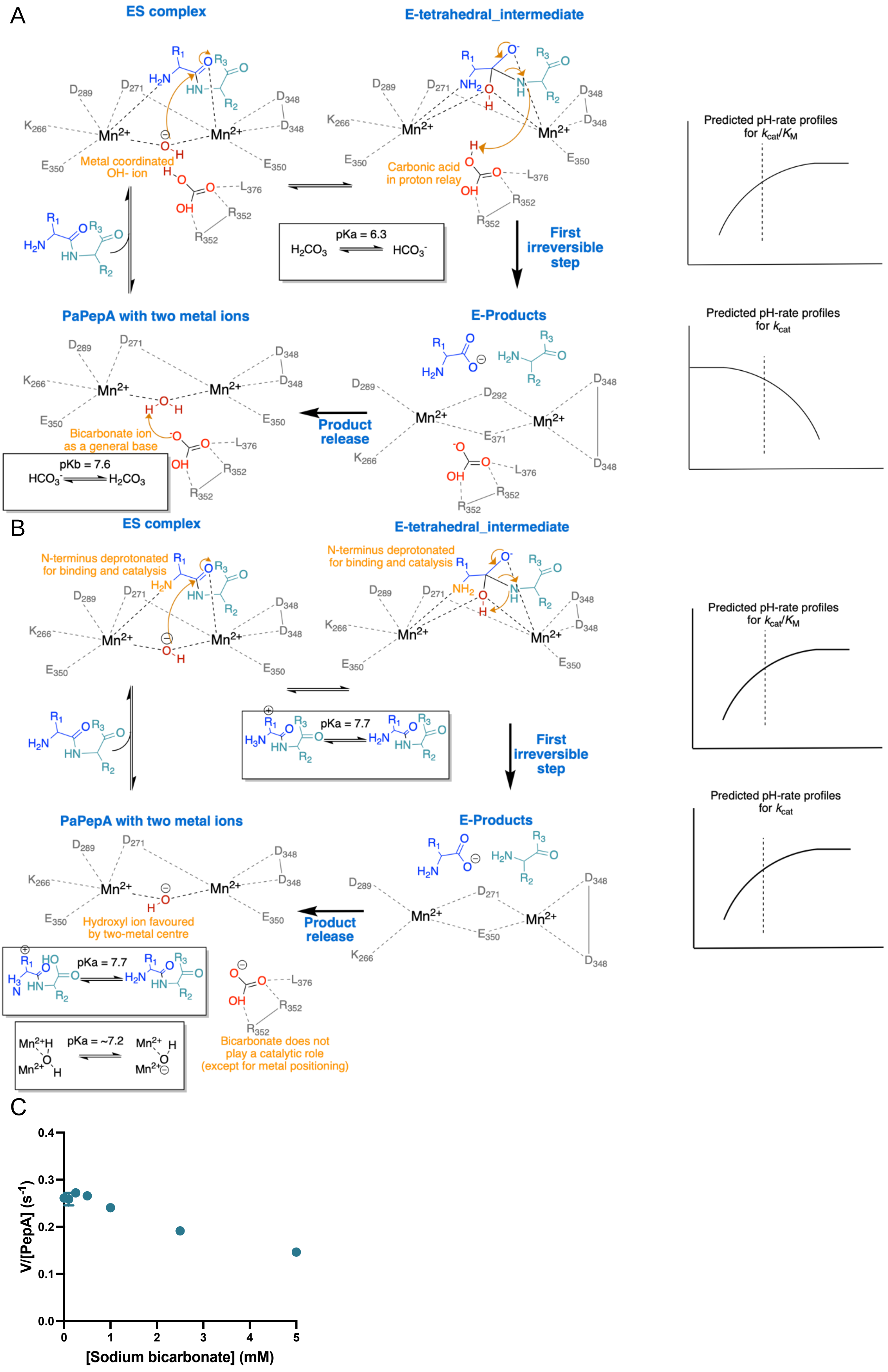
A
ES complex
E-tetrahedral_intermediate
Metal coordinated OH- ion
Carbonic acid in proton relay
pKa = 6.3
First irreversible step
PaPepA with two metal ions
E-Products
Product release
Bicarbonate ion as a general base
pKb = 7.6
Predicted pH-rate profiles for k_{cat}/K_M
Predicted pH-rate profiles for k_{cat}
B
ES complex
E-tetrahedral_intermediate
N-terminus deprotonated for binding and catalysis
pKa = 7.7
First irreversible step
PaPepA with two metal ions
E-Products
Product release
Hydroxyl ion favoured by two-metal centre
pKa = 7.7
pKa = ~7.2
Bicarbonate does not play a catalytic role (except for metal positioning)
Predicted pH-rate profiles for k_{cat}/K_M
Predicted pH-rate profiles for k_{cat}
C
V/[PepA] (s^{-1})
[Sodium bicarbonate] (mM)

Figure S9: Catalytic mechanism proposals for PaPepA A) Mechanism accounting for bicarbonate acting as a catalytic base to activate metal-bound water, and taking part as carbonic acid in proton shuttle enabling dissolution of the tetrahedral intermediate. B) Mechanism proposed here, in which the metal coordinated water can give rise to the pKa observed for k_{cat}/K_M, while deprotonated peptide N-terminus could contribute to k_{cat} profile. On the right of both mechanism there is a scheme of expected pH-rate profiles for each mechanism. C) Leu-pNA cleavage assays to investigate potential activation by bicarbonate. Assays carried out in the presence of excess Leu-pNA and $MnCl_2$ using degassed enzyme, buffers, and assay stock solutions, and increasing concentrations of sodium bicarbonate. Data are mean for three replicates ± SEM

A

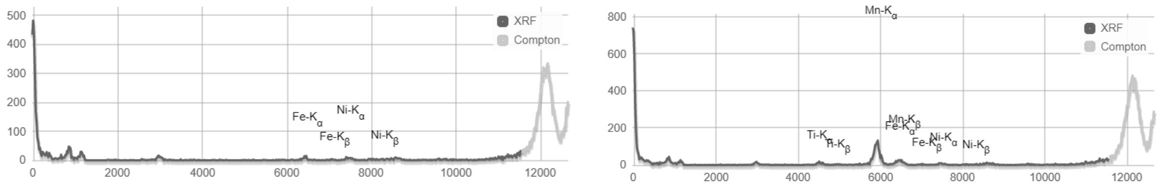

B

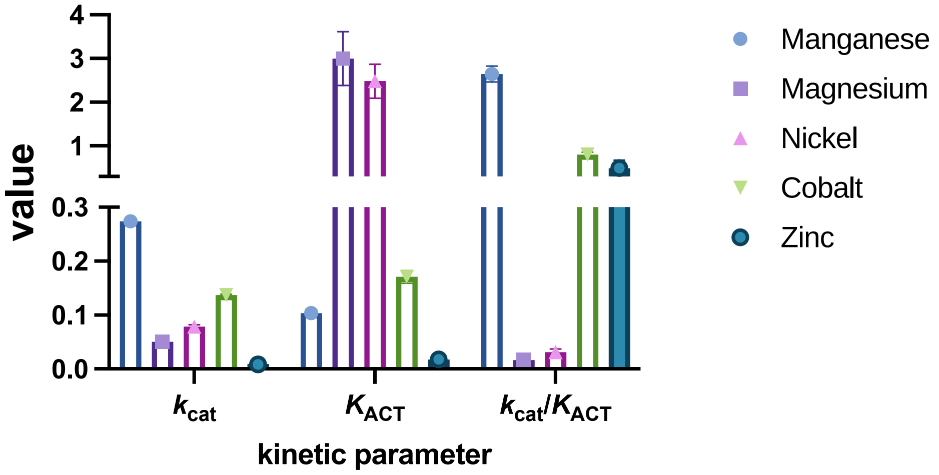

Figure S10: *PaPepA metal binding.* A) Crystal fluorescence edge scan. Left: Multichannel analyser (MCA) spectrum of *Pa*PepA immediately after purification, showing no bound metal (left). Right: vs MCA spectrum of *Pa*PepA co-crystallised with 5 mM $MnCl_2$ (right). B) Summary of kinetic parameters using different metal ions as activators of *Pa*PepA.

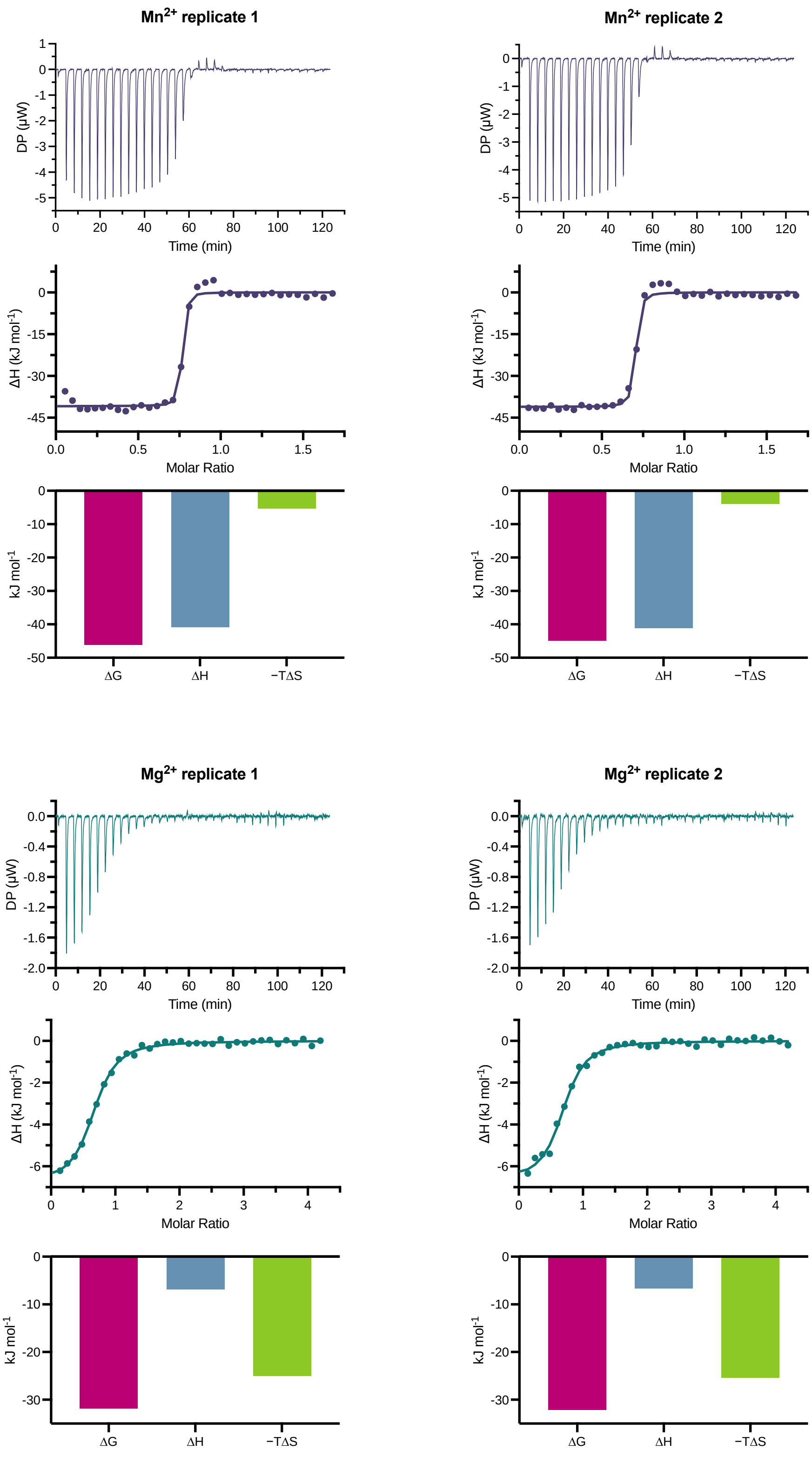

Figure S11: Isothermal titration calorimetry (ITC) of PaPepA with Mn2+ and Mg2+. Duplicates are shown, for each curve top is raw data, middle is fit to a one binding site model, bottom are the fitted and calculated values for ΔG, ΔH and -TΔS.

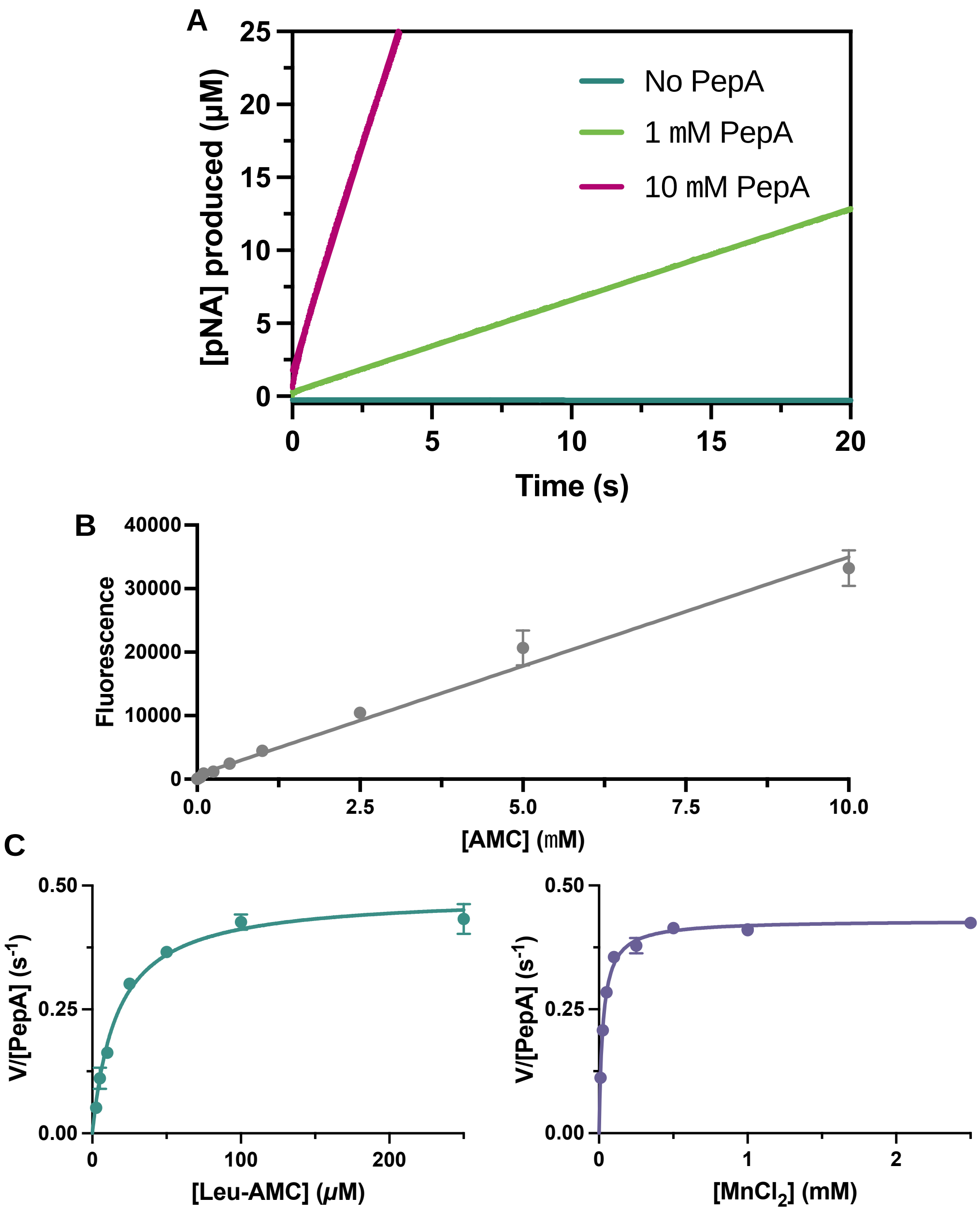

Figure S12: A) Multiple turnover stopped flow experiment with excess Leu-pNA. (Leu-pNA present at 250 μM). B) Calibration curve of fluorescence readout as a function of AMC concentration for plate reader-based assays evaluating Leu-AMC as a *Pa*PepA substrate. C) Initial velocity plotted as a function of substrate concentration fit to a Michaelis-Menten curve. (left) $MnCl_2$ added in excess, (right) Leu-AMC added in excess.

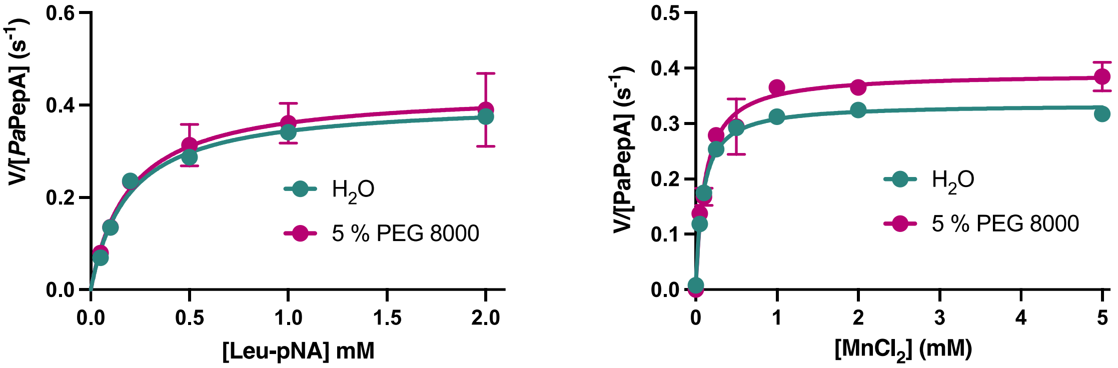

	H_2O	5 % PEG 8000
k_{cat}	0.335 ± 0.004	0.391 ± 0.008
K_M	0.0869 ± 0.005	0.11 ± 0.01
k_{cat}/K_M	3.9 ± 0.2	3.5 ± 0.4

	H_2O	5 % PEG 8000
k_{cat}	0.409 ± 0.009	0.43 ± 0.02
K_{ACT}	0.19 ± 0.01	0.19 ± 0.03
k_{cat}/K_{ACT}	2.2 ± 0.2	2.2 ± 0.4

Figure S13: Solvent macroviscosity studies for Leu-pNA cleavage reaction catalysed by *Pa*PepA. Varying concentration of [Leu-pNA] (*left*) or $MnCl_2$ (*right*) in 0% PEG-8000 (teal) and 5% PEG-8000 (pink). Data represent mean ± SE from triplicate measurements, and are fit to the Michaelis Menten equation. The table shows the effect of PEG-8000 on steady-state rate constants and represent value ± SEM.

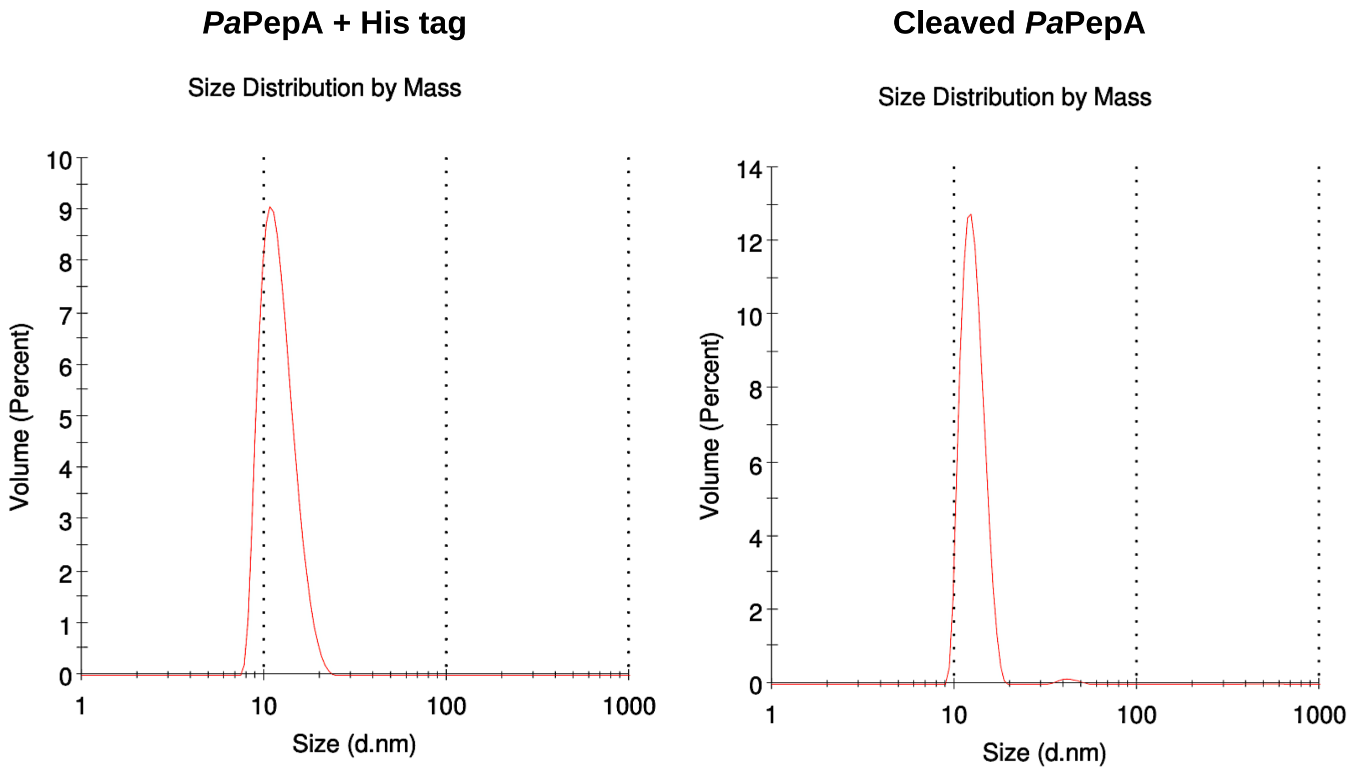

	His tagged	Cleaved
Monomer MW (kDa)	54.99	52.43
Estimated MW (kDa) mean ± SD	302.2 ± 69.5	283.3 ± 58.5

Figure S14: Dynamic Light Scattering of PaPepA: Hydrodynamic diameters of His-tagged and cleaved *Pa*PepA species. Data weighted by particle volumes. Estimated MWs (assuming globular protein) and their associated errors are reported.

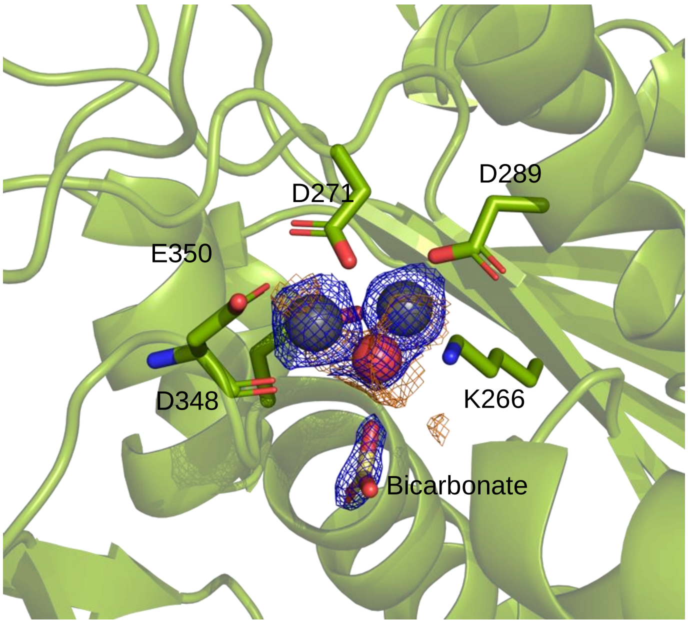

Figure S15: Mn2+ binding site. 2Fo-Fc map (blue) and Fo-Fc map (orange) depicting two Mn binding sites and the bridging water molecule. Mn ions are coloured in grey, water molecule is red.

Supplementary Tables

Table S1: Primers for PaPepA cloning (Gibson assembly) and site directed mutagenesis.

Primer name	Sequence
Backbone_F	acgtgccaagTGAAAGCTTCCCCCTAGC
Backbone_R	agaattccatCCCGCCCTGGAAATACAAATTTTCAG
PepA gBlock_F	tttccaggggATGGAATTCTTGGTAAAGAGTGTG
PepA gBlock_R	tttccagggcgggATGGAATTCTTGGTAAAGAGTGTG
PepA_D289A_F	AATGAAATTTgcgATGTGTGGTGCAGC
PepA_D289A_R	TCATCCATACCCAGGCCT
PepA_D348A_F	TCTGAACACAgcgGCAGAAGGCC
PepA_D348A_R	ATTTCCACCGTTTGTCCAC

Table S2: pNA calibration curves at different pHs. Values are slopes for change in absorbance in function of pNA concentration at different pH values using a mixed buffer (100 mM of MES, CHES, and HEPES).

pH	pNA (ΔA_{405} μM^{-1})
6.5	1.62×10^{-2}
7.0	1.56×10^{-2}
7.5	1.47×10^{-2}
8.0	1.61×10^{-2}
8.5	1.68×10^{-2}

Table S3: ADH standard peptides following digestion with Trypsin. Singly, doubly, triply and quadruply protonated charge states were searched for. In bold = peptides which would result from full trypsin cleavage of ADH. However, up to three missed cleavages were allowed in our search parameters. Peptides in bold contain 0 missed cleavages following trypsin digestion.

Molecule Name	Formula	Adduct	Charge	m/z	Cleaved?
SIPETQK	**$C_{34}H_{59}N_9O_{13}$**	**[M+H]<br>[M+2H]<br>[M+3H]**	**1<br>2<br>3**	**802.431<br>401.719<br>268.149**	**Digested**
SIPETQKGVIFYESHGK	$C_{87}H_{134}N_{22}O_{27}$	[M+2H]<br>[M+3H]<br>[M+4H]	2<br>3<br>4	960.497<br>640.667<br>480.753	Not digested
**GVIFYESHGK**	**$C_{53}H_{77}N_{13}O_{15}$**	**[M+H]<br>[M+2H]<br>[M+3H]**	**1<br>2<br>3**	**1136.573<br>568.790<br>379.529**	**Digested**
GVIFYESHGKLEYK	$C_{79}H_{116}N_{18}O_{22}$	[M+2H]<br>[M+3H]<br>[M+4H]	2<br>3<br>4	835.433<br>557.291<br>418.220	Digested
LEYKDIPVPKPK	$C_{68}H_{111}N_{15}O_{18}$	[M+2H]<br>[M+3H]<br>[M+4H]	2<br>3<br>4	713.919<br>476.282<br>357.463	Not digested
**DIPVPKPK**	**$C_{42}H_{72}N_{10}O_{11}$**	**[M+H]<br>[M+2H]**	**1<br>2**	**893.545<br>447.276**	**Not digested**
PKANELLINVK	$C_{56}H_{99}N_{15}O_{16}$	[M+H]<br>[M+2H]<br>[M+3H]	1<br>2<br>3	1238.747<br>619.878<br>413.588	Digested
**ANELLINVK**	**$C_{45}H_{80}N_{12}O_{14}$**	**[M+H]<br>[M+2H]<br>[M+3H]**	**1<br>2<br>3**	**1013.599<br>507.303<br>338.538**	**Digested**
**YSGVC[+57.0]HTDLHAWHGDWPLPVK**	**$C_{114}H_{159}N_{31}O_{30}S$**	**[M+2H]<br>[M+3H]<br>[M+4H]**	**2<br>3<br>4**	**1238.087<br>825.727<br>619.547**	**Digested**
**LPLVGGHEGAGVVVGMGENVK**	**$C_{88}H_{147}N_{25}O_{27}S$**	**[M+2H]<br>[M+3H]<br>[M+4H]**	**2<br>3<br>4**	**1010.038<br>673.695<br>505.523**	**Not digested**
**IGDYAGIK**	**$C_{38}H_{61}N_9O_{12}$**	**[M+H]<br>[M+2H]**	**1<br>2**	**836.451<br>418.729**	**Digested**
**VLGIDGGEGK**	**$C_{40}H_{69}N_{11}O_{15}$**	**[M+H]<br>[M+2H]**	**1<br>2**	**944.505<br>472.756**	**Digested**

		**[M+3H]**	**3**	**315.506**	
VLGIDGGEGKEELFR	$C_{71}H_{115}N_{19}O_{24}$	[M+2H]<br>[M+3H]<br>[M+4H]	2<br>3<br>4	809.925<br>540.286<br>405.466	Digested
**SIGGEVFIDFTK**	$C_{61}H_{93}N_{13}O_{19}$	**[M+2H]**<br>**[M+3H]**<br>**[M+4H]**	**2**<br>**3**<br>**4**	**656.843**<br>**438.231**<br>**328.925**	**Digested**
EKDIVGAVLK	$C_{48}H_{86}N_{12}O_{15}$	[M+H]<br>[M+2H]<br>[M+3H]	1<br>2<br>3	1071.641<br>536.324<br>357.885	Not digested
**DIVGAVLK**	$C_{37}H_{67}N_{9}O_{11}$	**[M+H]**<br>**[M+2H]**	**1**<br>**2**	**814.503**<br>**407.755**	**Not digested**
**ATDGGAHGVINVSVSEAAIEASTR**	$C_{95}H_{158}N_{30}O_{37}$	**[M+2H]**<br>**[M+3H]**<br>**[M+4H]**	**2**<br>**3**<br>**4**	**1156.577**<br>**771.387**<br>**578.792**	**Not digested**
**ANGTTVLVGMPAGAK**	$C_{59}H_{103}N_{17}O_{19}S$	**[M+2H]**<br>**[M+3H]**<br>**[M+4H]**	**2**<br>**3**<br>**4**	**693.874**<br>**462.919**<br>**347.441**	**Not digested**
**C[+57.0]C[+57.0]SDVFNQVVK**	$C_{56}H_{90}N_{16}O_{19}S_2$	**[M+2H]**<br>**[M+3H]**<br>**[M+4H]**	**2**<br>**3**<br>**4**	**678.308**<br>**452.541**<br>**339.658**	**Digested**
**SISIVGSYVGNR**	$C_{54}H_{90}N_{16}O_{18}$	**[M+2H]**<br>**[M+3H]**<br>**[M+4H]**	**2**<br>**3**<br>**4**	**626.338**<br>**417.895**<br>**313.673**	**Digested**
**EALDFFAR**	$C_{45}H_{65}N_{11}O_{13}$	**[M+H]**<br>**[M+2H]**<br>**[M+3H]**	**1**<br>**2**<br>**3**	**968.484**<br>**484.745**<br>**323.499**	**Digested**
GLVKSPIK	$C_{39}H_{72}N_{10}O_{10}$	[M+H]<br>[M+2H]	1<br>2	841.551<br>421.279	Not digested
SPIKVVGLSTLPEIYEK	$C_{87}H_{145}N_{19}O_{26}$	[M+2H]<br>[M+3H]<br>[M+4H]	2<br>3<br>4	937.038<br>625.028<br>469.022	Not digested
**VVGLSTLPEIYEK**	$C_{67}H_{110}N_{14}O_{21}$	**[M+2H]**<br>**[M+3H]**<br>**[M+4H]**	**2**<br>**3**<br>**4**	**724.406**<br>**483.273**<br>**362.707**	**Digested**
**YVVDTSK**	$C_{36}H_{58}N_{8}O_{13}$	**[M+H]**<br>**[M+2H]**	**1**<br>**2**	**811.420**<br>**406.213**	**Digested**

Table S4: SIR Mass list and cone voltages

Compound	Mass (m/z)	Cone (V)
K-NH_2 + (H^+)	146.1	30
K-NH_2 + (Na^+)	168.1	30
KK-NH_2 + (H^+)	274.2	30
KK-NH_2 + (Na^+)	296.2	30
RKK-NH_2 + (H^+)	430.3	30
RKK-NH_2 + (Na^+)	452.3	30
FRKK-NH_2 + (H^+)	577.4	30
FRKK-NH_2 + (Na^+)	599.4	30
GFRKK-NH_2 + (H^+)	634.4	30
GFRKK-NH_2 + (Na^+)	656.4	30
SGFRKK-NH_2 + (H^+)	721.4	30
SGFRKK-NH_2 + (Na^+)	743.4	30
QSGFRKK-NH_2 + (H^+)	849.5	30
QSGFRKK-NH_2 + (Na^+)	871.5	30
LQSGFRKK-NH_2 + (H^+)	962.6	30
LQSGFRKK-NH_2 + (Na^+)	984.6	30
VLQSGFRKK-NH_2 + (H^+)	1061.7	30
VLQSGFRKK-NH_2 + (Na^+)	1083.6	30
AVLQSGFRKK-NH_2 + (H^+)	1132.7	30
AVLQSGFRKK-NH_2 + (Na^+)	1154.7	30

Table S5: LC-MS - QDa mass detector settings:

Solvent manager:
Solvent A: Water + 0.01% formic acid
Solvent B: Acetonitrile

HPLC gradient

Time (min)	Flow rate (mL min^{-1})	% A	% B
0	0.4	99	1.0
2		99	1.0
9		1.0	99
11		1.0	99
11.1		99	1.0
14		99	1.0

UV Detector:
Wavelengths: 220 nm and 260 nm
Sampling Rate: 1 points sec^{-1}

Autosampler
Wash Solvent Name: 50% Water/50% Acetonitrile
Purge Solvent Name: 50% Water/50% Acetonitrile
Target Sample Temperature: 10.0 C
Target Column Temperature: 40.0 C
Injection Volume (μL) - 10.00

QDa settings:

Instrument Parameters - Function 1:

Polarity	ES+
Capillary (kV)	0.80
Cone (V) (With Ramping)	30.00
Source Temperature (°C)	120
Probe Temperature (°C)	600

Instrument Parameters - Function 2:

Polarity	ES+
Calibration	Static 2
Capillary (kV)	0.80
Cone (V)	30
Source Temperature (°C)	120
Probe Temperature (°C)	600

Table S6: Exponential fitted data for time courses with AVLQSGFRKK-NH_2

Concentration (µM)-peptide	k1	k2	Equation fitted
200-AVLQSGFRKK	0.022	31.8	Single Exponential
100-AVLQSGFRKK	0.050	13.7	Single Exponential
50-AVLQSGFRKK	0.049	14.1	Single Exponential
25-AVLQSGFRKK	0.048	14.5	Single Exponential
200-VLQSGFRKK	0.039	0.037	Double Exponential
100-VLQSGFRKK	0.089	0.086	Double Exponential
50-VLQSGFRKK	0.094	0.092	Double Exponential
25-VLQSGFRKK	0.077	0.074	Double Exponential
200-LQSGFRKK	0.030	0.029	Double Exponential
100-LQSGFRKK	0.073	0.071	Double Exponential
50-LQSGFRKK	0.076	0.074	Double Exponential
25-LQSGFRKK	0.036	0.035	Double Exponential
200-QSGFRKK	0.016	0.016	Double Exponential
100-QSGFRKK	0.045	0.044	Double Exponential
50-QSGFRKK	0.046	0.045	Double Exponential
25-QSGFRKK	NA	NA	Double Exponential
200-SGFRKK	0.008		Single Exponential
100-SGFRKK	0.023		Single Exponential
50-SGFRKK	0.026		Single Exponential
25-SGFRKK	0.012		Single Exponential
200-GFRKK	25.800		Linear regression
100-GFRKK	49.360		Linear regression
50-GFRKK	45.800		Linear regression
25-GFRKK	18.610		Linear regression
200-FRKK	1.289		Linear regression
100-FRKK	2.540		Linear regression
50-FRKK	2.581		Linear regression
25-FRKK	0.968		Linear regression
100-RKK	2.326		Linear regression
50-RKK	1.421		Linear regression

Table S7: Average binding constants, concentrations, and stoichiometries from ITC

Data for Mn^{2+} and Mg^{2+} Binding to *Pa*PepA.

Metal	[PepA] (μM)	[Metal] (μM)	N	K_D (nM)
	35.80 ± 1.5	400.0	1	12.2 ± 1.5
**$MnCl_2$**	47.00	526.5 ± 22.5	1	16.30 ± 0.01
	47.00	400.0	0.76 ± 0.03	12.2 ± 0.1
	32.60	1000	1	1845 ± 182
**$MgCl_2$**	48.00	1470	1	2720 ± 470
	48.00	1000	0.679 ± 0.001	1845 ± 182

Table S8: Slopes when varying viscogen (sucrose) concentrations

	**Slope (sucrose)**
**k_{cat}**	0.14 ± 0.05
**k_{cat}/K_{ACT} (Mn^{2+} variation)**	-0.10 ± 0.06
**k_{cat}**	0.03 ± 0.01
**k_{cat}/K_M (Leu-pNA variation)**	-0.06 ± 0.02

Table S9: SKIEs calculated by fitting proton inventory data (Figure 5C) to distinct models.

**Model fitted**		**ϕ1**	**Φ2**	**Φsolvent**	**Calculated SKIE**	**Experimental SKIE**
1 TS proton $V_n/V_0 = (1 - n + n * \varphi_1)$	$k_{cat\text{-}Mn}$	0.080 ± 0.02			12.5 ± 3.6	3.9 ± 0.1
	$k_{cat\text{-}Mg}$	0.19 ± 0.02			5.2 ± 0.7	3.1 ± 0.1
1TS + solvent contribution $V_n/V_0 = (1 - n + n * \varphi_1) * {\varphi_s}^n$	$k_{cat\text{-}Mn}$	0.25 ± 0.09		0.69 ± 0.12	5.6 ± 2.3	
	$k_{cat\text{-}Mg}$	0.6 ± 0.3		0.50 ± 0.19	1.6 ± 1.0	
2 TS same φ $V_n/V_0 = (1 - n + n * \varphi_1)^2$	$k_{cat\text{-}Mn}$	0.44 ± 0.01			5.1 ± 0.1	
	$k_{cat\text{-}Mg}$	0.53 ± 0.01			3.7 ± 0.1	
2 TS different φ $V_n/V_0 = (1 - n + n * \varphi_1) * (1 - n + n * \varphi_2)$	$k_{cat\text{-}Mn}$	0.6 ± 0.4	0.3 ± 0.3		5.3 ± 5.7	
	$k_{cat\text{-}Mg}$	0.52 ± large	>1000		1.9 ± large	
2 TS same φ + solvent contribution $V_n/V_0 = (1 - n + n * \varphi_1)^2 * {\varphi_s}^n$	$k_{cat\text{-}Mn}$	0.41 ± 0.07		1.1 ± 0.3	5.4 ± 1.6	
	$k_{cat\text{-}Mg}$	0.7 ± 0.2		0.6 ± 0.3	3.2 ± 2.1	

SKIE calculated based on the equation below, where φ^{RS} stands for reactant state fractionation factor, φ^{TS} stands for transition state fractionation factor:

$$^{D2O}k = \prod_i^x \varphi_i^{RS} \Big/ \prod_i^x \varphi_i^{TS}$$

, it should be mentioned that this calculated SKIE is an intrinsic solvent kinetic isotope effect, calculated on Supporting note 4. Additionally, models accounting for reactant state protons did not converge to fitted values. Proton inventories on $^{D2O}k_{cat}/K_{M\text{-}LeupNA\text{-}Mn}$ and $^{D2O}k_{cat}/K_{ACT}$

Had errors that exceeded 20% and therefore were not interpreted mechanistically.

Calculation of intrinsic Solvent kinetic isotope effects

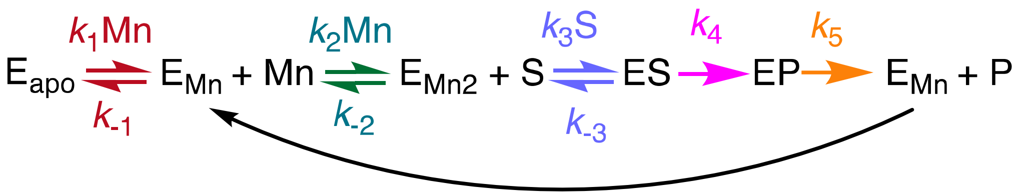

With the scheme above and the fitted rate constants according to the table below:

	value
***k*1** (µM^{-1}s^{-1})	86.5 ± 0.1
***k*-1** (s^{-1})	1.18*
***k*2** (µM^{-1}s^{-1})	18.9 ± 0.1
***k*-2** (s^{-1})	1.89*
***k*3** (µM^{-1}s^{-1})	1.58 ± 0.01
k-3 (s^{-1})	25.3*
***k*4** (s^{-1})	6.27 ± 0.01
***k*5** (s^{-1})	13.4 ± 0.08

*calculated values

$$^{D2O}\left(\frac{kcat}{KM - Leu}\right) = \frac{^{D2O}k4 + cf}{1 + cf}$$

$$cf = \frac{k_4}{k_{-3}}$$

$$^{D2O}V = \frac{^{D2O}k_4 + c_{vf}}{1 + c_{vf}}$$

c_{vf} = ratio of catalysis = $^{k_4}/_{k_5}$

$$^{D2O}V = \frac{^{D2O}k_4 + ^{k_4}/_{k_5}}{1 + ^{k_4}/_{k_5}}$$

$^{D2O}k_{cat\text{-}Mn}$ = 3.9 = ($^{D2O}k_4$ + 6.24/13.4)/(1 + 6.24/13.4) => $^{D2O}k_4$ = 5.2, c_{vf} = 0.5
$^{D2O}k_{cat}/K_{M\text{-}LeupNA\text{-}Mn}$ = 2.2 = ($^{D2O}k_4$+ 6.24/25.3)/(1+6.24/25.3) => $^{D2O}k_4$ = 2.4, c_f = 0.13

$^{D2O}k_4$ is used to simplify nomenclature, but the rate constant k_4 is a macroscopic rate constant , likely including several microscopic steps with some contributing to $k_{cat}/K_{M\text{-}LeupNA\text{-}Mn}$ and others to k_{cat}, hence different values for calculated intrinsic solvent kinetic isotope effects.

Table S10: Crystallographic data

	8PZY - **Hexameric *Pa*PepA bound to Mn^{2+}**	*8PZO* - **Apo *Pa*PepA (monomer)**	*8PZM* - **Bestatin bound *Pa*PepA (monomer)**
**Resolution range**	52.06 - 1.97 (2.04 - 1.97)	45.49 - 1.8 (1.864 - 1.8)	43.73 - 1.7 (1.761 - 1.7)
**Space group**	P 21 21 21	P 63 2 2	P 63 2 2
**Unit cell**	87.53 183.03 316.53 90 90 90	181.95 181.95 87.43 90 90 120	182.05 182.05 87.57 90 90 120
**Total reflections**	714934 (70896)	157637 (15519)	3770747 (364951)
**Unique reflections**	357487 (35452)	78823 (7762)	93574 (9225)
**Multiplicity**	2.0 (2.0)	2.0 (2.0)	40.3 (39.6)
**Completeness (%)**	99.94 (99.94)	99.94 (99.63)	99.98 (99.98)
**Mean I/sigma(I)**	10.65 (0.81)	18.18 (0.93)	13.35 (0.63)
**Wilson B-factor**	40.64	33.95	26.27
**R-merge**	0.03428 (0.8347)	0.01654 (0.7219)	0.2685 (5.309)
**R-meas**	0.04848 (1.18)	0.02339 (1.021)	0.272 (5.377)
**R-pim**	0.03428 (0.8347)	0.01654 (0.7219)	0.04285 (0.8506)
**CC1/2**	0.999 (0.444)	1 (0.475)	0.999 (0.347)
**CC***	1 (0.784)	1 (0.803)	1 (0.718)
**Reflections used in refinement**	357296 (35432)	78803 (7749)	93570 (9225)
**Reflections used for R-free**	17937 (1733)	3873 (364)	4726 (462)
**R-work**	0.1765 (0.3512)	0.1724 (0.3326)	0.1683 (0.3008)
**R-free**	0.2040 (0.3718)	0.1926 (0.3413)	0.1856 (0.3137)
**CC(work)**	0.969 (0.694)	0.970 (0.719)	0.972 (0.669)
**CC(free)**	0.967 (0.641)	0.962 (0.653)	0.961 (0.650)

**Number of non-hydrogen atoms**	24697	**Number of non-hydrogen atoms**	4231	**Number of non-hydrogen atoms**	4438
**macromolecules**	22298	**macromolecules**	3732	**macromolecules**	3722
**ligands**	301	**ligands**	91	**ligands**	125
**solvent**	2270	**solvent**	455	**solvent**	657
**Protein residues**	2994	**Protein residues**	500	**Protein residues**	499
**RMS(bonds)**	0.008	**RMS(bonds)**	0.007	**RMS(bonds)**	0.008
**RMS(angles)**	0.97	**RMS(angles)**	0.92	**RMS(angles)**	0.97
**Ramachandran favored (%)**	97.72	**Ramachandran favored (%)**	98.59	**Ramachandran favored (%)**	97.99
**Ramachandran allowed (%)**	2.15	**Ramachandran allowed (%)**	1.41	**Ramachandran allowed (%)**	1.81
**Ramachandran outliers (%)**	0.13	**Ramachandran outliers (%)**	0	**Ramachandran outliers (%)**	0.2
**Rotamer outliers (%)**	0.35	**Rotamer outliers (%)**	0	**Rotamer outliers (%)**	0
**Clashscore**	5.48	**Clashscore**	4.56	**Clashscore**	6.37
**Average B-factor**	44.81	**Average B-factor**	42.15	**Average B-factor**	30.78
**macromolecules**	44.06	**macromolecules**	40.91	**macromolecules**	28.85
**ligands**	54.41	**ligands**	55.85	**ligands**	37.56
**solvent**	51.63	**solvent**	51	**solvent**	41.1

References

(1) Cleland, W. W. Partition Analysis and the Concept of Net Rate Constants as Tools in Enzyme Kinetics. *Biochemistry* **1975**, *14*(14), 3220-3224.

(2) Gadda, G.; Fitzpatrick, P. F. Solvent Isotope and Viscosity Effects on the Steady-State Kinetics of the Flavoprotein Nitroalkane Oxidase. *FEBS Lett* **2013**, *587*(17), 2785-2789.
